# Non-bioenergetic roles of mitochondrial GPD2 promote tumor progression

**DOI:** 10.7150/thno.75973

**Published:** 2023-01-01

**Authors:** Sehyun Oh, Sihyang Jo, Martina Bajzikova, Han Sun Kim, Thien T. P. Dao, Jakub Rohlena, Jin-Mo Kim, Jiri Neuzil, Sunghyouk Park

**Affiliations:** 1 College of Pharmacy, Natural Product Research Institute, Seoul National University, Seoul 08826, Korea.; 2 School of Pharmacy and Medical Science, Griffith University, Southport, Qld, Australia.; 3 Institute of Physiology, Czech Academy of Sciences, Prague, Czech Republic.; 4 Institute of Biotechnology, Czech Academy of Sciences, Prague-West, Czech Republic.; 5 Faculty of Science, Charles University, Prague, Czech Republic.

**Keywords:** cancer, mitochondria, GPD2, DHAP, ether lipids

## Abstract

**Rationale:** Despite growing evidence for mitochondria's involvement in cancer, the roles of specific metabolic components outside the respiratory complex have been little explored. We conducted metabolomic studies on mitochondrial DNA (mtDNA)-deficient (ρ0) cancer cells with lower proliferation rates to clarify the undefined roles of mitochondria in cancer growth.

**Methods and results:** Despite extensive metabolic downregulation, ρ0 cells exhibited high glycerol-3-phosphate (G3P) level, due to low activity of mitochondrial glycerol-3-phosphate dehydrogenase (GPD2). Knockout (KO) of GPD2 resulted in cell growth suppression as well as inhibition of tumor progression *in vivo.* Surprisingly, this was unrelated to the conventional bioenergetic function of GPD2. Instead, multi-omics results suggested major changes in ether lipid metabolism, for which GPD2 provides dihydroxyacetone phosphate (DHAP) in ether lipid biosynthesis. GPD2 KO cells exhibited significantly lower ether lipid level, and their slower growth was rescued by supplementation of a DHAP precursor or ether lipids. Mechanistically, ether lipid metabolism was associated with Akt pathway, and the downregulation of Akt/mTORC1 pathway due to GPD2 KO was rescued by DHAP supplementation.

**Conclusion:** Overall, the GPD2-ether lipid-Akt axis is newly described for the control of cancer growth. DHAP supply, a non-bioenergetic process, may constitute an important role of mitochondria in cancer.

## Introduction

Mitochondria are multi-functional organelles that act as a power plant for eukaryotic cells, and are also responsible for critical biological processes such as cell proliferation 1, reactive oxygen species (ROS) production 2, and metabolism 3-5. Ever since Otto Warburg proposed that cancer cells have impaired mitochondrial respiration and therefore rely on glycolysis even in the presence of oxygen 6, 7, the “aerobic glycolysis” or “Warburg effect” has dominated the cancer metabolism field. For the last 10 years, however, numerous studies have provided evidence that mitochondria are essential for tumorigenesis and tumor progression. The current consensus is that cancer cells feature plasticity in the use of mitochondrial respiration and glycolysis 8, 9. Nevertheless, our understanding of direct tumorigenic metabolism of mitochondrial proteins has remained mostly limited to components of well-known bioenergetic functions such as tricarboxylic acid (TCA) cycle and oxidative phosphorylation (OXPHOS), or to epigenetic effects of oncogenic mutations in succinate dehydrogenase (SDH), fumarate hydratase (FH), or isocitrate dehydrogenase (IDH) 10, 11. Therefore, studies on effects of other mitochondrial proteins on cancer growth should expand our understanding of mitochondria's involvement in cancer.

GPD2 is an enzyme encoded by a single *GPD2* gene located on chromosome 2q24.1 of the human genome and is located at the outer surface of the inner mitochondrial membrane 12. Its enzymatic activity entails oxidation of G3P to the glycolytic intermediate DHAP and transfer of electrons to flavin adenine dinucleotide (FAD), which, in turn, conveys them to coenzyme Q (CoQ) in the respiratory chain 13. Although any reaction can run in forward or backward direction in theory, it is well-established that the GPD2 reaction runs in a direction that generates DHAP in physiological conditions. As GPD2 is located within mitochondria, specifically in the intermembrane space (IMS), the transport of G3P and DHAP between the IMS and cytosol is thought to be conducted by β‑barrel protein voltage-dependent anion channel (VDAC) on the mitochondrial outer membrane 14, 15. Conversion of G3P to DHAP is one half of the reaction of the glycerol phosphate shuttle (GPS) that transports cytosolic NADH to mitochondrial FADH_2_, the other half being the reverse reaction mediated by cytosolic glycerol-3-phosphate dehydrogenase (GPD1). These functions render GPD2 an important link in both glucose-lipid metabolism and glycolysis-OXPHOS interfaces. First, formation of glycolytic DHAP from G3P catalyzed by GPD2 can affect biosynthetic lipogenesis, since G3P is the substrate of the rate-limiting enzyme for phospholipid and triglyceride (TG) biosynthesis, viz. glycerol-3-phosphate acyltransferase (GPAT) 16. Second, participation of GPD2 in the electron transport chain (ETC) can regulate bioenergetic formation of ATP and generation of ROS 17-19. This is epitomized by GPD2 being a target for suppression of gluconeogenesis by metformin 20 in a redox-dependent manner 21, GPD2 regulating gluconeogenesis in the liver starting from glycerol 22, 23, and also being involved in retrograde electron transport to inhibit inflammatory responses 24. There also have been several studies indicating the contribution of GPD2 to cancer. Among these findings, upregulated GPD2 in thyroid cancer enhanced the rate of OXPHOS in support of cancer cell growth 25; proliferation of glioma cells was increased due to higher glycolytic rate upon GPD2 activation 26, and anchorage-independent cell growth was inhibited by GPD2 knockdown due to disrupted energy metabolism in liver cancer cells 27. Thus, our understanding of the role of GPD2 in cancer growth is mostly related to its 'conventional' bioenergetic functions in glycolysis and OXPHOS.

Ether lipids are types of glycerophospholipids where the hydrocarbon chain is attached to an ether bond at the *sn-1* position of the glycerol backbone 28, 29. They are a major component of the cell membrane 30-32, constituting about 20% of phospholipids in mammals 33-35, with the highest content found in the brain and heart 36, 37. There are two types of ether lipids, plasmanyl- and plasmenyl-phospholipids (plasmalogens), the biosynthesis of which starts from the conversion of G3P to DHAP by GPD2. Addition of a fatty alcohol chain occurs in peroxisomes by a series of enzymes involving glyceronephosphate O-acyltransferase (GNPAT) 38, alkylglycerone phosphate synthase (AGPS) 39, and fatty acyl-CoA reductase 1 (FAR1) 40, 41, whereas the addition of an acyl chain occurs in the endoplasmic reticulum 41. Ether lipids are elevated in various cancer cell lines 42, 43 and tumors 44-46, and increased levels of ether lipids in breast cancer and colon cancer patients have been reported 47, 48. Downregulation of ether lipids by AGPS knockdown in cancer cells has been linked to reduced cancer aggressiveness 43, and correlation between ether lipids and cancer metastasis has been described 49. While these studies suggest involvement of ether lipids in cancer, there is little understanding of the underlying metabolism by which they regulate cancer. Similarly, even with the diverse biological functions of ether lipids 37, the causality between their elevation and cancer proliferation has not been well established 50.

In this study, by applying metabolomics, transcriptomics, and biochemical approaches using mtDNA-deficient, ρ0, and GPD2 KO cells, we uncovered a novel, non-bioenergetic role of mitochondrial GPD2 that is linked to ether lipids' cancer-cell-growth and tumor-progression-related functions.

## Methods

### Cell preparation

The 4T1 murine mammary carcinoma cell line was purchased from ATCC (Manassas, VA, USA). The production of the 4T1-derived mtDNA-deficient ρ0 cell line and its mitochondria-restored sublines (D5, D10, D15, D20, D25, and D60) were described previously 51, 52.

The CRISPR-Cas9 system was used for the preparation of GPD2 genetic KO in the 4T1 cell line. The pSp-U6-Cas9-2A-Puro vectors, containing the target guide RNA sequence and Cas9 nuclease expressing cassette, were purchased from Macrogen (Seoul, Korea). Different guide RNA sequences were used for producing each GPD2 KO cell line: (1) 5'-TCAGGTGAGCCTGGCATATGTGG-3'; (2) 5'-GCACTAGATGCCGTCACCAGAGG-3'. The 4T1 cells were transiently transfected with the CRISPR-Cas9 vector and the Lipofectamine™ 3000 Transfection Reagent (Cat# L3000001, Thermo Fisher Scientific, Waltham, MA, USA) for 24 h. After the transfection, the cells were selected with 5 µg/mL puromycin (Cat# P8833, Sigma-Aldrich, St. Louis, MO, USA) for over 72 h to eliminate the untransfected cells. The surviving cells after puromycin selection were single-cell cultured in 96-well plates, and the single-cell-derived clones were obtained from each well. The confirmation of successful GPD2 KO was determined by the loss of GPD2 protein expression in Western blot analysis.

The overexpression of GPD2 in 4T1 cells was mediated by transfecting Gpd2 (BC021359) Mouse Untagged Clone (Cat# MC206608, OriGene Technologies, Inc., Rockville, MD, USA) with Lipofectamine™ 3000 Transfection Reagent following the manufacturer's instructions. After 24 h, the cells were selected with 400 µg/mL Geneticin™ (Cat# 10131035, Thermo Fisher Scientific) for 48 h to eliminate the untransfected cells. The surviving cells after Geneticin selection were harvested for Western blot and Liquid Chromatography-Mass Spectrometry (LC-MS) analysis.

The 4T1 and derived sublines were grown in high-glucose RPMI medium (Cat# LM 011-51, Welgene, Gyeongsan, Korea) containing 10% FBS (Cat# S 001-07, Welgene) and 1% penicillin streptomycin (Cat# LS 203-01, Welgene) with additional supplementation of 50 mg/mL uridine (Cat# U3003, Sigma-Aldrich). All of the cell lines were cultured at 37 °C in a 5% CO_2_ humidified incubator.

Human breast cancer cell line (MDA-MB-231), human pancreatic cancer cell line (AsPC-1), and human liver cancer cell lines (Huh-7, HepG2, SK-HEP-1, and PLC/PRF/5) were purchased from Korean Cell Line Bank (Seoul, Korea). These cell lines were cultured in DMEM medium (Cat# LM 001-05, Welgene) containing 10% FBS and 1% penicillin streptomycin.

### Cell proliferation assay

The growth rates of 4T1 and 4T1 ρ0 cells were measured by seeding the same number of cells to a 100 mm dish and incubating for 5 days in 4T1 growth medium. The cells were then detached from the dish by trypsin-EDTA (Cat# LS 015-10, Welgene), and the number of cells was counted with the Countess II FL automated cell counter (Invitrogen). For comparison of the growth rates of 4T1 and 4T1 GPD2 KO cells, the same procedure was conducted except that the RPMI medium (Cat# LM 011-01, Welgene) containing 10% FBS and 1% penicillin streptomycin was used. The rest of the experiments in our study were performed in this growth medium unless indicated otherwise.

GPD2 inhibitor, KM04416 (Cat# 002-906-717, Molport, Beacon, NY, USA), was prepared in 100 mM stock in DMSO. The effect of KM04416 on the cell growth of 4T1 and 4T1 ρ0 cells was observed at the final concentrations of 0, 5, 10, and 20 µM. For the control group (0 µM), it was treated with the same volume of DMSO. The cell numbers were counted after 48 h. The human cancer cell lines (MDA-MB-231, AsPC-1, Huh-7, HepG2, SK-HEP-1, and PLC/PRF/5) were treated with 20 µM KM04416, at which concentration the inhibitor exhibited significantly different cell growth between 4T1 and 4T1 ρ0 cells.

For the cell growth rescue experiment, 2 mM DHA (Cat# D5818, TCI, Portland, OR, USA) or 100 µM plasmalogen PC (18:0p/18:1) (Cat# 852467C, Avanti Polar Lipids, Birmingham, AL, USA) was supplemented to the 4T1 GPD2 KO cells. The plasmalogen PC (18:0p/18:1), as dissolved in chloroform, was prepared in a liposome form before treatment to the cells. The plasmalogen in chloroform was evaporated by dry-nitrogen stream, and the dried lipid film was kept frozen until hydrated and used for the experiment. The hydration of dried lipid film was accomplished by simply mixing lipid with growth medium and agitating the mixture for 1 h at room temperature. Afterward, the glass vial containing the lipid mixture was suspended in a bath sonicator for 10 min of sonication. The growth medium containing the liposomes with the final concentration of 100 µM was supplemented to the cells. Either DHA or plasmalogen were treated to the cells only once at the 0-hour point, and the cell numbers were counted after 72 h.

### Clonogenic assay

For the clonogenic assay, 50 cells were seeded onto 6-well plates. The cells were grown for 14 days in RPMI medium (Cat# LM 011-01, Welgene) containing 10% FBS and 1% penicillin streptomycin. After the colonies were formed, the medium was rinsed with DPBS, and the colonies were fixed and stained with a mixture of 0.5% crystal violet staining solution. The crystal violet mixture was removed after 30 min, and rinsed carefully with tap water. The plates with colonies were dried at room temperature before the imaging.

### Wound healing assay

The cells were seeded onto 24-well plates, and grown in RPMI medium (Cat# LM 011-01, Welgene) containing 10% FBS and 1% penicillin streptomycin for 24 h until it forms confluent cell monolayer. Next, the monolayers were incubated with growth medium containing 1% FBS for 16 h before making a scratch with SPLScar 24-Well Scrather (Cat# 201924, SPL Lifesciences, Pocheon, Korea). After scratching, the monolayers were incubated for 48 h with growth medium containing 1% FBS to observe the wound healing. The images were taken using a light microscope.

### Nuclear magnetic resonance (NMR) spectroscopy

The 4T1 and 4T1 ρ0 cells were incubated overnight in Gibco glucose-free DMEM medium (Cat# 11966-025, Thermo Fisher Scientific) containing 10% Gibco dialyzed FBS (Cat# 26400-044, Thermo Fisher Scientific) and 1% penicillin streptomycin, with 5 mM D-Glucose (U-13C6, 99%) (Cat# CLM-1396, Cambridge Isotope Laboratories, Inc., Andover, MA, USA). The cells were washed with DPBS and lysed with 200 mL of 5:3:2 methanol/acetonitrile/water mixture. The lysates were centrifuged at 27,000 x g, 4 °C for 20 min, and the supernatant was separated into a new tube and evaporated by a centrifugal evaporator. The dried pellets were then mixed with 500 µL NMR buffer (2 mM Na_2_HPO_4_ and 5 mM NaH_2_PO_4_ in D_2_O with 0.025% trimethylsilylpropionic acid sodium salt-d4) for sample preparation. The procedure of lipid extraction for TG and phosphatidylcholine (PC) is described below in the LC-MS analysis section. The prepared samples were transferred to a 5 mm NMR tube, and the 2D spectrum of the sample was obtained by ^1^H-^13^C Heteronuclear Single Quantum Coherence (HSQC) measured with an 800 MHz Bruker Avance III HD spectrometer equipped with a 5 mm CPTCI CryoProbe (Bruker BioSpin, Rheinstetten, Germany) at College of Pharmacy, Seoul National University. The spectral width was set to 12 ppm for ^1^H and 40 ppm for ^13^C, and the time domain was 2048 (^1^H) x 200 (^13^C) with the number of scans set to 16. The O1P was 4.7 ppm, and O2P, 27 ppm. The acquired NMR spectrum was analyzed with Topspin 3.6.2 software provided by Bruker.

### LC-MS analysis

For the extraction of metabolites and lipids from the cells, the same number of cells were seeded onto 100 mm dishes and incubated with growth medium. After 24 h, the cultured medium was collected for measurement of extracellular lactate, and the cells were washed with DPBS and lysed with a 2:1 methanol/chloroform mixture (total volume: 600 mL) for the extraction. The lysates were vortexed and incubated in liquid nitrogen for 60 s. The frozen samples were thawed at room temperature, and then mixed with an additional 1:1 chloroform/water mixture (total volume: 400 mL). Next, the samples were centrifuged at 15,000 x g, 4 °C for 20 min for the phase separation. After centrifugation, the upper methanol and water phase containing metabolites and the lower chloroform phase containing dissolved lipids were separated into different sample tubes and dried with a centrifugal evaporator. The dried samples were kept at -80 °C until used.

The standard compounds of DHAP, G3P, ATP, ADP, and lactate were purchased from Sigma-Aldrich, and plasmalogen PC (18:0p/18:1) was obtained from Avanti Polar Lipids (Cat# 852467) for the MRM set-up. The dried cell extract samples were diluted in 1:1 acetonitrile/water mixture for sample injection into the LC-MS. The Agilent 1290 Infinity LC System (Agilent, Santa Clara, CA, USA) and BEH amide column (1.7 µm, 100 × 2.1 mm; Cat# 186004801, Waters, Milford, MA, USA) were used for the chromatography, with 20 mM ammonium acetate (pH 9.0 by ammonium hydroxide) and acetonitrile for the mobile phases. The Agilent Technologies 6460 Triple Quad LC-MS at College of Pharmacy, Seoul National University was used for the detection of DHAP, G3P, ATP, ADP, and lactate in negative ion mode. The same Agilent LC-MS hardware with Kinetex C18 column (2.6 µm, 100 x 4.6 mm; Cat# 00D-4462-E0, Phenomenex, Torrance, CA, USA), and 10 mM ammonium formate with 0.1% formic acid in 6:4 acetonitrile/water mixture (pH 9.0 by ammonium hydroxide) and 10 mM ammonium formate with 0.1% formic acid in 9:1 isopropyl alcohol/water mixture for the mobile phases were applied to detect plasmalogen PC (18:0p/18:1) in positive ion mode. MassHunter software (Agilent) was used for the MS data analysis. The signal intensity value of MRM obtained from LC-MS was normalized by the paired cellular BCA protein assay value measured with the Pierce BCA protein assay kit (Cat# 23227, Thermo Fisher Scientific).

For the untargeted MS analysis of lipids in the 4T1 and 4T1 GPD2 KO (1) cell lines, the extracted lipid samples were analyzed by Q Exactive Plus Hybrid Quadrupole-Orbitrap Mass Spectrometer (Thermo Fisher Scientific) and Vanquish UHPLC system (Thermo Fisher Scientific) at College of Pharmacy, Seoul National University, with the same column and buffers used as for the detection of plasmalogen PC (18:0p/18:1). The MS1 and MS2 spectra were obtained in the full scan range of *m/z* 100-1200 in negative ion mode. The acquired data were analyzed by MS-DIAL software (RIKEN, Yokohama, Japan) for lipid annotation. The identified lipid species were ranked by p-value, and the top 20 species were listed on a heatmap. The heatmap was drawn with the “heatmaply” package 53 in R and GraphPad Prism v9.3.0. The Q1 and Q3 values of each ether lipid species were obtained from MS-DIAL software for the MRM assay detection.

### Western blot analysis

The cells were lysed in RIPA buffer with additional protease inhibitor cocktail (Cat# ab271306, Abcam, Cambridge, England, UK) and phosphatase inhibitor cocktail (Cat# ab201112, Abcam). The lysates were centrifuged at 15,000 x g, 4 °C for 30 min, and only the supernatants were collected without cell debris. Then, the supernatants were mixed with 5x laemmli sample buffer. The prepared samples were loaded onto 10% SDS-PAGE gel and transferred to a PVDF or nitrocellulose membrane. The membrane was blocked with 5% bovine serum albumin and then incubated with antibodies. The antibodies used in the experiment are as follows: β-Actin (Cat# sc-47778, Santa Cruz Biotechnology, Inc., Dallas, TX, USA, RRID: AB_2714189); GPD1 (Cat# sc-390379, Santa Cruz Biotechnology, Inc.); GPD2 (Cat# sc-393620, Santa Cruz Biotechnology, Inc.); GNPAT (Cat# MBS129275, MyBioSource, San Diego, CA, USA); FAR1 (Cat# MBS3209766, MyBioSource); PI3K (Cat# sc-7174, Santa Cruz Biotechnology, Inc., RRID: AB_2252476); PDK1 (Cat# 3062, Cell Signaling Technology, Danvers, MA, USA, RRID: AB_2236832); Phospho-PDK1 (Cat# AP0477, ABclonal, Woburn, MA, USA, RRID: AB_2771409); PTEN (Cat# 9552, Cell Signaling Technology, RRID: AB_10694066); AKT1 (Cat# 1081-1, Antibodypedia, Houston, TX, USA, RRID: AB_562035,); Phospho-Akt (Thr308) (Cat# 4056, Cell Signaling Technology, RRID: AB_331163); AKT1 (phospho S473) (Cat# ab81283, Abcam, RRID: AB_2224551); mTOR (Cat# 2972, Cell Signaling Technology, RRID: AB_330978); mTOR (phospho S2448) (Cat# ab109268, Abcam, RRID: AB_10888105); p70 S6K (Cat#9202, Cell Signaling Technology, RRID: AB_331676); Phospho-p70 S6 Kinase (Thr389) (Cat# 9205, Cell Signaling Technology, RRID: AB_330944); Catalase (Cat# sc-271803, Santa Cruz Biotechnology, Inc., RRID: AB_10708550); GPx3 (Cat# ab27325, Abcam, RRID: AB_2112263); SOD-2 (Cat# sc-30080, Santa Cruz Biotechnology, Inc., RRID: AB_661470); Goat anti-Mouse IgG (H+L) Secondary Antibody, HRP (Cat# 31430, Invitrogen); Goat anti-Rabbit IgG (H+L) Secondary Antibody, HRP (Cat# 31460, Invitrogen). Imaging of blots by chemiluminescence was conducted using the ImageQuant LAS 4000 biomolecular imager (GE Healthcare, Chicago, IL, USA).

### GPD2 enzyme activity test

To detect GPD2 activity of the cells, the mitochondria were isolated as follows. The cells were first dissolved in 1 mL hypotonic buffer (10 mM HEPES, 10 mM KCl, and 1.5 mM MgCl_2_ with pH 7.9) with 10 µL 10% TERGITOL solution (Cat# NP40S, Sigma-Aldrich), 20 µL PMSF (Cat# 11359061001, Roche, Basel, Switzerland), 10 µL pepstatin A (Cat# 516481, Sigma-Aldrich), 4 µL aprotinin (Cat# A1153, Sigma-Aldrich), and 10 µL DTT (Cat# 10708984001, Roche). Next, the sample was mixed vigorously with a syringe, and centrifuged at 2,000 x g, 4 °C for 5 min. The supernatant was separated, and centrifuged again at 8,000 x g, 4 °C for 10 min to acquire the mitochondria pellet at the bottom of the tube. The collected mitochondria were resuspended in an assay buffer (50 mM KCl, 1 mg/mL BSA, 10 mM Tris-HCl, 1 mM EDTA, and 1 mM KCN with pH 7.4), and aliquoted into a 96-well plate with 50 μM cytochrome c (Cyt c) (Cat# C7752, Sigma-Aldrich). The detection of GPD2 activity was initiated by the addition of 20 mM glycerophosphate (Cat# L03425, Alfa Aesar, Haverhill, MA, USA) before the data acquisition by VersaMax microplate reader (Molecular Devices, San Jose, CA, USA). The enzyme activity by the reduction of Cyt c was detected by measuring the absorbance at 550 nm for every 5 min at 30 °C. The enzyme activity was calculated by subtracting the control background (cells without glycerophosphate treatment).

### Animal experiment

Six-week-old female BALB/c mice were supplied by Central Lab. Animal Inc. (Seoul, Korea). All animal housing and experiment procedures were performed under the institutional guidelines and ethical authorization approved by Seoul National University Institutional Animal Care and Use Committee (approval # SNU-190812-1). The mice were acclimatized to the housing for one week prior to the experiment. The 4T1 and 4T1 GPD2 KO (1) cells were diluted in DPBS, and mixed with Matrigel (Cat# 354248, Corning, Corning, NY, USA) (1:1). The cell-matrigel mixture was injected subcutaneously into the right flank of the mice. After the injection, there was one week of standby to allow for solid-tumor formation. After one week, the tumor size was measured once per week with a caliper. The tumor-size calculation equation was as follows: tumor size (mm^3^) = [length (mm) × width (mm) × height (mm)] π/6.

### CCK-8 assay

The same number of cells were seeded onto 96-well plates, and incubated for 6 h for cell adhesion. After the cells were attached to the plate, 10 µL of CCK-8 solution (Cat# CCK-3000, DonginLS, Seoul, Korea) was added to each well of the plate. The plate was incubated for 2 h in the incubator. The absorbance was measured at 450 nm using a microplate reader.

### Respiration measurement

The chambers of the Oroboros Oxygraph-2k instrument (Oroboros Instruments, Innsbruck, Austria) were calibrated at 37 °C with the respiration medium (Mir05 medium: 0.5 mM EGTA, 3 mM MgCl2, 60 mM K-lactobionate, 20 mM taurine, 10 mM KH2PO4, 110 mM sucrose, 1 g/L essentially fatty acid-free BSA, 20 mM Hepes, pH 7.1 at 30 °C). Cells were collected by trypsinization, washed with PBS, resuspended in the respiration medium, and transferred to the chamber of the Oroboros Oxygraph-2k instrument. After oxygen signal stabilization, the chamber was closed, and routine respiration was recorded. For Complex I (CI), Complex II (CII), and GPD2-mediated respiration, digitonin-permeabilized cells were used in all of the experiments (10 µg digitonin per million cells.). The CI-mediated respiration was assessed by adding 10 mM glutamate, 2 mM malate, 3 mM ADP, and 10 µM Cyt c. CII- and GPD2-mediated respiration were determined in the presence of 0 mM succinate or glycerol-3-phosphate, respectively, 0.5 μM rotenone, 3 mM ADP, and 10 μM Cyt c. The maximal respiration in the uncoupled state was then achieved by carbonyl cyanide m-chlorophenyl hydrazone (CCCP) titration in 0.5 μM steps. Respiration rates after 2.5 μM antimycin A addition were subtracted to obtain the mitochondria-specific rates.

### Mitochondrial ATP measurement

The cells were seeded onto 6-well plates, and incubated for 24 h. A final concentration of 10 µM BioTracker™ ATP-Red Live Cell Dye (Cat# SCT045, EMD Millipore Corporation, Temecula, CA, USA) was treated to the cells in culture. After 30 min, the cells were trypsinized and resuspended in DPBS for the data acquisition (Excitation/Emission = 510/570 nm) by BD FACSCalibur™ (BD Biosciences, Franklin Lakes, NJ, USA) at College of Pharmacy, Seoul National University. The acquired data were analyzed with BD FlowJo™ Software (BD Biosciences) by calculating percentage of positive population compared to unstained negative control.

### ROS measurement

The cells were seeded onto 96-well black plates (Cat# 3603, Corning) and incubated overnight. The final concentration of 5 µM H2DCFDA (Cat# D399, Invitrogen) or 2 µM MitoSOX Red Mitochondrial Superoxide Indicator (Cat# M36008, Invitrogen) was treated to the cells. The plate was incubated for 45 min for staining with light blocking. The fluorescence (Excitation/Emission = 492/527 nm for H2DCFDA, 510/580 nm for MitoSOX Red) was measured by a SpectraMAX M5 micro plate reader (Molecular Devices). The final fluorescence value was calculated by control background subtraction (wells without H2DCFDA or MitoSOX Red dye).

### RNA sequencing (RNA-Seq) analysis

Total RNA was extracted from the cells using an Easy-Spin Total RNA Extraction Kit (Cat# 17221, iNtRON Biotechnology Inc., Seongnam, Korea). After sample quality control, the RNA samples were processed for library construction according to the TruSeq Stranded mRNA Sample Preparation Guide (Part # 15031047 Rev. E). For the quality of data on Illumina sequencing platforms, the libraries were quantified by qPCR according to the Illumina qPCR Quantification Protocol Guide. The sequencing of the cDNA fragments made from the RNA was conducted using the NovaSeq 6000 system (Illumina, San Diego, CA, USA) with a paired-end read length of 101. Prior to the data analysis, the adapter sequence was removed by the Trimmomatic 0.38 program. The bases that did not qualify for the window size of 4, mean quality of 15, and base quality less than 3 from the read's ends were eliminated. The trimmed data were created by removing reads shorter than the minimum length of 36 bp. For the reads that had undergone the pre-processing, the HISAT2 (version 2.1.0) program was used for the reference genome mapping with genomic DNA reference UCSC mm10. Transcript assembly was performed through the StringTie (version 1.3.4d) program with the reference-based aligned reads information. The FPKM (Fragments Per Kilobase of transcript per Million mapped reads) value was used for the expression profile. A further DEG (Differentially Expressed Genes) analysis was performed with the significant DEG results that had been selected under the following conditions: |fc|>=2 & exactTest raw p-value<0.05. With the significant gene lists, a gene-set enrichment analysis (GSEA) was conducted based on the KEGG (Kyoto Encyclopedia of Genes and Genomes) database (http://www.genome.jp/kegg/). Raw RNA-Seq data have been uploaded to NCBI's Gene Expression Omnibus (https://www.ncbi.nlm.nih.gov/geo/) and are accessible using GEO Series accession number GSE197257.

### DHAP supplementation

DHAP precursor, DHA, was used for the DHAP supplementation. The cells were seeded onto 100 mm cell culture dishes, and incubated for 24 h. Next, the cells were treated with 5 mM DHA in 5 mL RPMI medium (Cat# LM 011-01, Welgene) containing 10% FBS and 1% penicillin streptomycin for 120 min. After the incubation, the cells were extracted and sample-prepared for Western blot and LC-MS analysis to measure DHAP and ether lipid level, and to detect PI3K/Akt pathway components.

### Lipid rafts isolation

The cells were grown in 100 mm cell culture dishes, and the lipid rafts were isolated with Minute^TM^ Plasma Membrane-Derived Lipid Raft Isolation Kit (Cat# LR-042, Invent Biotechnologies, Inc., Plymouth, MN, USA) following the manufacturer's instructions. The isolated lipid rafts were diluted in RIPA buffer as described in Western blot analysis section. For the determination of the lipid rafts-recruited Akt level, the lipid rafts samples were equally loaded in SDS-PAGE gel compared to the whole cell lysate.

### Cell cycle analysis

The cells were seeded onto 6-well plates, and incubated for 24 h. Harvested cells were fixed in 70% ethanol, and incubated at -20 °C for 1 h. The cell pellets were collected by centrifugation (2,000 rpm/4 °C/5 min), and the collected cells were stained with 0.1 mL PI/RNase Staining Buffer (Cat# 550825, BD Biosciences) with 0.8 μg RNase for 15 min at room temperature. The stained cells were resuspended in DPBS before the assay. BD FACSCalibur™ (BD Biosciences) was used to acquire flow cytometry data. The acquired data were analyzed with BD FlowJo™ Software (BD Biosciences). The result was represented in the percentage of cell numbers in each phases of the cell cycle.

### Bioinformatics analysis

For the comparison of *GPD2* gene expression between the cancer and normal tissues, the gene expression dataset from Xena (TCGA TARGET GTEx cohort, dataset: gene expression RNAseq - RSEM expected_count (DESeq2 standardized), version 2018-05-08, (https://xenabrowser.net) was downloaded, and the classification of matched tumor-normal pairs proceeded as described previously 54. Violin plots were drawn with GraphPad Prism v9.3.0. Survival curves were obtained from GEPIA 2 (http://gepia2.cancer-pku.cn) 55. Immunohistochemistry images of GPD2 protein expression in tissues were obtained from The Human Protein Atlas (http://www.proteinatlas.org) 56.

### Statistical analysis

Data were obtained from three biologically independent samples unless indicated otherwise. The graphs were drawn in mean values with standard deviations as determined by the GraphPad Prism v9.3.0 program. The statistical significance between groups was assessed by two-tailed unpaired Student's t-test, with a p-value of less than 0.05 being regarded as significant unless stated otherwise. The “*” in the graphs indicates statistically significant difference (“*”: p < 0.05; “**”: p < 0.005; “***”: p < 0.0005), and “N.S.,” 'not significant.' The graphical abstract was created with BioRender.com.

## Results

### Mitochondrial damage causes loss of GPD2 activity leading to decreased DHAP/G3P ratio

**Figure 1:**
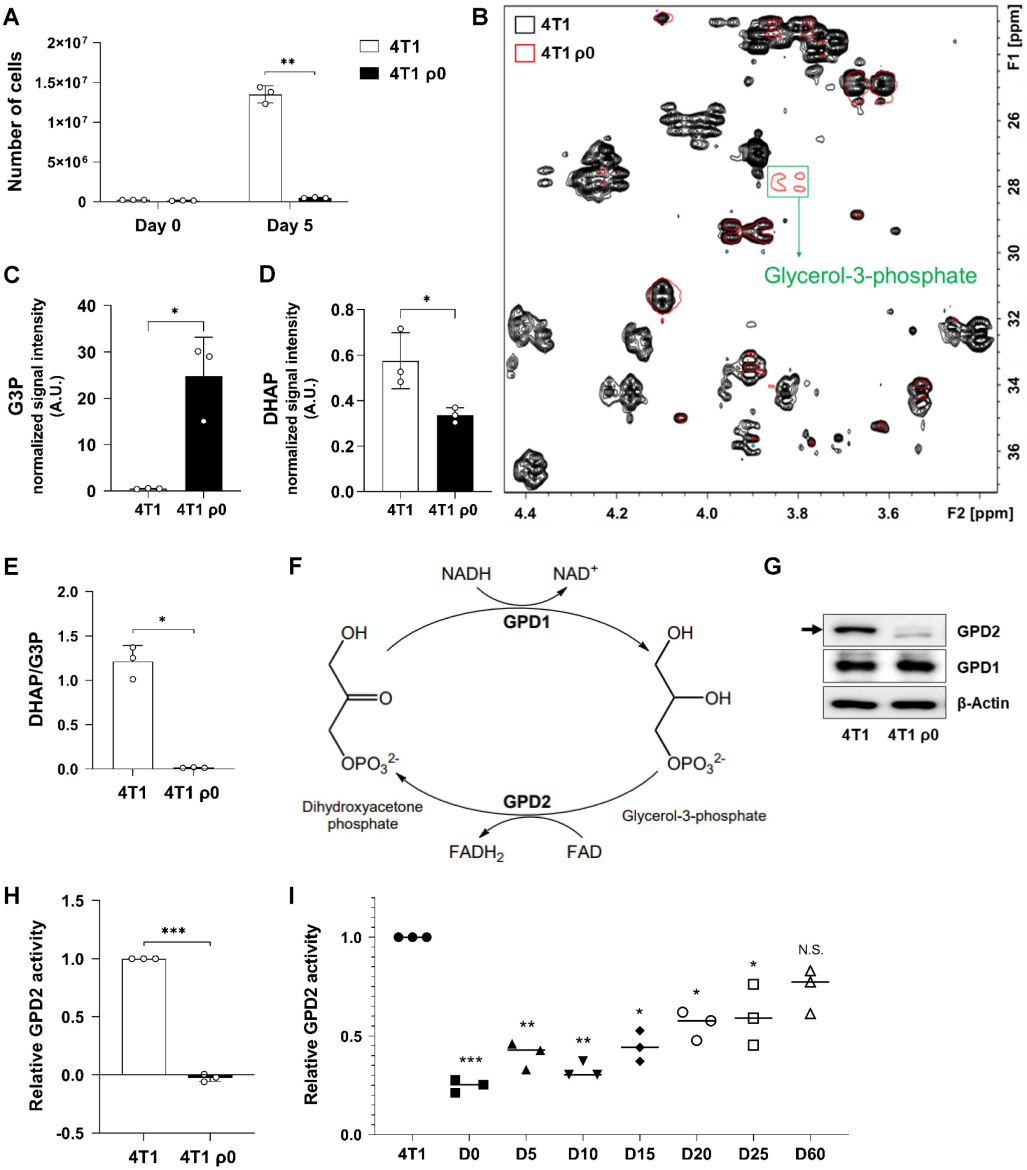
** mtDNA-deficient 4T1 ρ0 cells exhibit low DHAP/G3P ratio due to lack of GPD2 expression. (A)** Growth rates of 4T1 and 4T1 ρ0 cells by counting cell numbers in 5 days of incubation. **(B)** Comparison of representative ^1^H-^13^C HSQC-NMR spectra of metabolites from 4T1 and 4T1 ρ0 cells. The 2D spectra of 4T1 are marked in black, 4T1 ρ0 in red, and the G3P peak is indicated in a green box. **(C)** Level of G3P in 4T1 and 4T1 ρ0 cells. **(D)** Level of DHAP in 4T1 and 4T1 ρ0 cells. **(E)** Cellular DHAP/G3P ratio in 4T1 and 4T1 ρ0 cells. For data (C-E), the signal intensities of DHAP and G3P were measured by LC-MS. **(F)** Metabolism of glycerol phosphate shuttle that catalyzes interconversion between DHAP and G3P. **(G)** Protein expression of GPD1 and GPD2 in 4T1 and 4T1 ρ0 cells as detected by Western blot analysis. The arrow indicates the band size of GPD2. **(H)** Relative GPD2 enzyme activity in 4T1 and 4T1 ρ0 cells. The reduction of Cyt c was measured by the absorbance at 550 nm with a microplate reader. **(I)** Relative GPD2 enzyme activity in 4T1, 4T1 ρ0 (D0; “D-” refers to 'day'), and 4T1 ρ0-derived sub-lines exhibiting gradual mtDNA restoration (D5, D10, D15, D20, D25, and D60). Data were obtained from three biologically independent samples. The p-value was calculated by comparing the experimental group with 4T1 control group using a two-tailed unpaired Student's t-test. The “*” in the graphs indicates statistically significant difference (“*”: p < 0.05; “**”: p < 0.005; “***”: p < 0.0005), and “N.S.,” 'not significant.' A.U., arbitrary unit.

Cancer cells with damaged mitochondria, represented by murine breast cancer 4T1 cells devoid of mtDNA, ρ0 cells 52, were found to proliferate much slower than their parental counterparts **(Figure 1A).** To investigate the differences in cell proliferation within a metabolic context, we performed metabolomic profiling by ^1^H-^13^C HSQC NMR analysis. Comparison of each 2D NMR spectrum revealed that most of the detected metabolites are present at higher levels in parental cells, which indicates that 4T1 ρ0 cells feature suppressed metabolic activity. Interestingly, G3P was one of the few metabolites whose level was much higher in 4T1 ρ0 cells **(Figure 1B).** Since G3P is synthesized directly from a glycolytic intermediate, DHAP, we assessed the levels of G3P and DHAP by LC-MS. In parallel with the NMR result, the G3P level was higher **(Figure 1C),** and the DHAP level was lower in ρ^0^ cells **(Figure 1D).** As a result, the calculated DHAP/G3P ratio was much lower for ρ0 cells than for parental cells **(Figure 1E).** Conversion of DHAP to G3P is mediated by cytosolic GPD1, while mitochondrial GPD2 mediates the reverse reaction **(Figure 1F).** We therefore assessed expression of both GPD1 and GPD2 proteins, and found little difference in GPD1 expression in 4T1 and 4T1 ρ0 cells, while there was much lower level of GPD2 in ρ0 cells **(Figure 1G).** Consistently, enzyme activity of GPD2 was not detectable in mitochondria-damaged ρ0 cells **(Figure 1H).** As GPD2 is located at the mitochondrial inner membrane,** t**he absence of cristae by inner membrane damage in ρ0 cells, as proven in our previous study 52, should contribute to the loss of GPD2 activity in those cells. To further test the relationship between mitochondrial function and GPD2 activity, we took advantage of our previously prepared 4T1 ρ0-derived sub-lines with gradual recovery of mitochondrial function due to increased level of mtDNA (from ρ0 to parental 4T1 cell level). More specifically, these sub-lines were explanted from syngeneic tumors formed in mice from 4T1 ρ0 cells on different days post-grafting (from day 5 through to day 60) 52. Consistent with the increasing mtDNA level, we observed gradually elevated GPD2 activity in the sub-lines **(Figure 1I).** Our results suggest that mitochondrial damage leads to perturbation of DHAP/G3P ratio due to the loss of mitochondrial GPD2 activity, which may be linked to slower proliferation of ρ0 cancer cells.

### GPD2 deletion affects cancer cell growth and tumor progression

**Figure 2:**
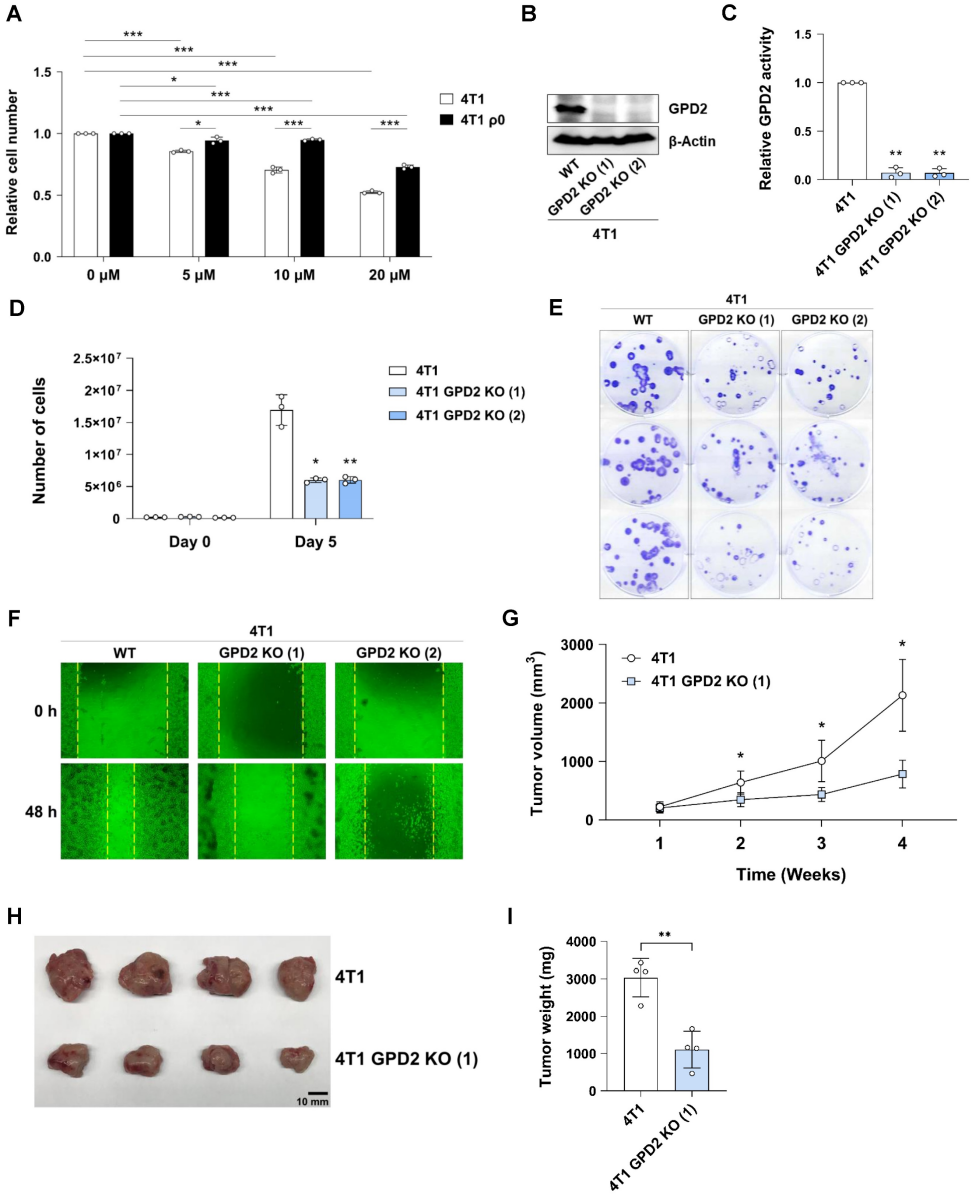
** Growth of 4T1 cancer cells *in vitro* and *in vivo* upon GPD2 modulation. (A)** Relative numbers of 4T1 and 4T1 ρ0 cells after treatment of KM04416 at different concentrations (0, 5, 10, and 20 µM) for 48 h. Both 4T1 and 4T1 ρ0 cell numbers were normalized by each paired control (0 µM KM04416). The cell numbers were counted with an automated cell counter. **(B)** Protein expression of GPD2 in 4T1 and 4T1 GPD2 KO cells as detected by Western blot analysis. **(C)** GPD2 enzyme activity in 4T1 and 4T1 GPD2 KO cells. **(D)** Growth rates of 4T1 and 4T1 GPD2 KO cells by counting cell numbers in 5 days of incubation. **(E)** Photographic image of clonogenic assay for 4T1 and 4T1 GPD2 KO cells. **(F)** Representative image of wound healing assay for 4T1 and 4T1 GPD2 KO cells (0 h and 48 h). **(G)** Tumor growth of syngeneic graft model of 4T1 and 4T1 GPD2 KO (1) cells. The size of the tumor was measured once every week with a caliper. **(H)** Photographic image of grafted tumors in (G). The tumors were extracted after 4 weeks, and photographed. **(I)** Weight of the extracted graft tumors in (H). Data were obtained from three biologically independent samples or four mice in each group unless indicated otherwise. The p-value was calculated by comparing the experimental group with 4T1 control group using a two-tailed unpaired Student's t-test. The “*” in the graphs indicates statistically significant difference (“*”: p < 0.05; “**”: p < 0.005; “***”: p < 0.0005).

To determine whether mitochondrial GPD2 has a causative role in cancer cell proliferation, we followed pharmacological and genetic approaches. First, a GPD2 inhibitor, KM04416 57, was treated to 4T1 and 4T1 ρ0 cells, where we observed a dose-dependent inhibitory effect, which was more pronounced in parental cells **(Figure 2A).** We next prepared 4T1 GPD2 KO cells by using CRISPR-Cas9 system, confirming the absence of GPD2 by protein expression **(Figure 2B)** and its activity **(Figure 2C)** in the two different KO clones. The 4T1 GPD2 KO cells exhibited much slower proliferation than 4T1 wildtype (WT) counterparts **(Figure 2D),** as well as less colony formation **(Figure 2E)** and lower migratory activity **(Figure 2F).** To assess the effect of GPD2 *in vivo*, we prepared syngeneic tumors by grafting 4T1 and 4T1 GPD2 KO cells into mice. Consistent with the *in vitro* result, the KO cell-derived tumors progressed significantly slower than tumors derived from their WT counterparts **(Figures 2G-I).** These results confirm the positive role of GPD2 in tumor progression, and inhibition of GPD2 may work as a therapeutic target for the cancer.

### Mitochondrial bioenergetics is not involved in the role of GPD2 in tumor progression

**Figure 3:**
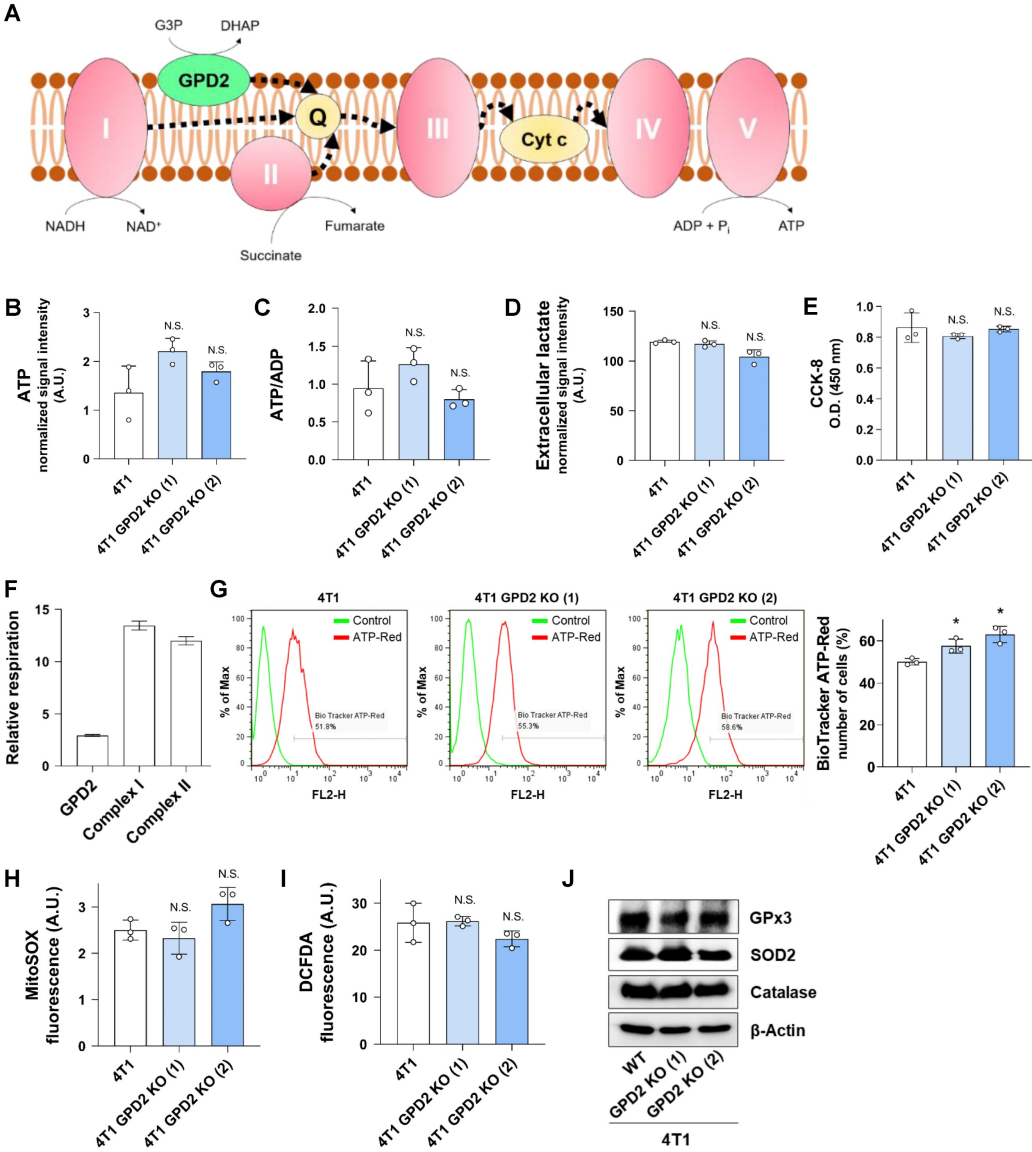
** Effect of GPD2 KO on mitochondrial respiration and bioenergetics. (A)** Scheme of electron flow in mitochondrial electron transport chain. CI, CII, and GPD2 transfer electrons to the Q pool, which contributes to ATP generation. Q: coenzyme Q. **(B)** Total cellular ATP level in 4T1 and 4T1 GPD2 KO cells measured by LC-MS. **(C)** Energy status of 4T1 and 4T1 GPD2 KO cells. The energy status was calculated from the ratio of ATP to ADP measured by LC-MS. **(D)** Lactate production of 4T1 and 4T1 GPD2 KO cells by measuring extracellular lactate in the cell-cultured growth medium by LC-MS. **(E)** CCK-8 assay of 4T1 and 4T1 GPD2 KO cells in the same number of cells. The value was measured by the absorbance at 450 nm with a microplate reader. **(F)** Relative level of GPD2, CI, or CII-dependent respiration in 4T1 cells as measured by high-resolution Oxygraph-2k respirometer. **(G)** Representative histogram and bar graph of mitochondrial ATP level in 4T1 and 4T1 GPD2 KO cells as measured by BioTracker ATP-Red Live Cell Dye with FACS analysis. **(H)** Mitochondrial superoxide level in 4T1 and 4T1 GPD2 KO cells as measured by MitoSOX fluorescence. **(I)** Total ROS level in 4T1 and 4T1 GPD2 KO cells as measured by DCFDA fluorescence. **(J)** Expression of antioxidant enzymes, catalase, GPx3, and SOD2 in 4T1 and 4T1 GPD2 KO cells, as detected by Western blot analysis. Data were obtained from three biologically independent samples. The p-value was calculated by comparing the experimental group with 4T1 control group by two-tailed unpaired Student's t-test. The “*” in the graphs indicates statistically significant difference (“*”: p < 0.05; “**”: p < 0.005; “***”: p < 0.0005), and “N.S.,” 'not significant.' A.U., arbitrary unit, O.D., optical density.

One of the well-studied roles of GPD2 is its contribution to the mitochondrial respiratory chain leading to ATP production **(Figure 3A).** Therefore, we investigated the effect of GPD2 KO on bioenergetics. We first assessed the total cellular ATP level and energy status in 4T1 and 4T1 GPD2 KO cells, observing no significant differences **(Figures 3B and C).** In addition, glycolytic activity, as indicated by lactate secretion, was similar in 4T1 and 4T1 GPD2 KO cells **(Figure 3D).** Overall mitochondrial dehydrogenase functionality was not different, either, based on the result from CCK-8 assay **(Figure 3E).** We assessed GPD2-dependent as well as CI- and CII-dependent respiration in WT cells. We found that GPD2-dependent respiration contributed only about 10% to the total respiration calculated as the sum of respirations from the three components **(Figure 3F).** To more specifically assess the ATP generation in mitochondria, we grew the WT and KO cells in galactose medium that allows the measurement of mitochondria-derived ATP 58. The ATP levels were not different according to the GPD2 KO status **(Figure S1).** Furthermore, when the mitochondrial ATP level was directly assessed with BioTracker ATP-Red Live Cell Dye, a dye that specifically targets ATP in mitochondria, the mitochondrial ATP level did not decrease in the KO cells **(Figure 3G).** These data confirm the above whole-cell level ATP results showing irrelevance of GPD2 in mitochondrial bioenergetics. We next evaluated ROS level, since mitochondrial respiration is the major source of superoxide formation 59. We found no significant differences both in mitochondrial superoxide **(Figure 3H),** and total ROS level **(Figure 3I)** between WT and GPD2 KO cells. Correspondingly, the level of antioxidant enzymes was not different **(Figure 3J).** These results indicate that GPD2 takes up only a small portion in mitochondrial respiration and affects cancer cell proliferation and tumor progression by a mechanism that is unrelated to mitochondrial bioenergetics.

### GPD2 regulates ether lipid synthesis to control cancer growth

**Figure 4:**
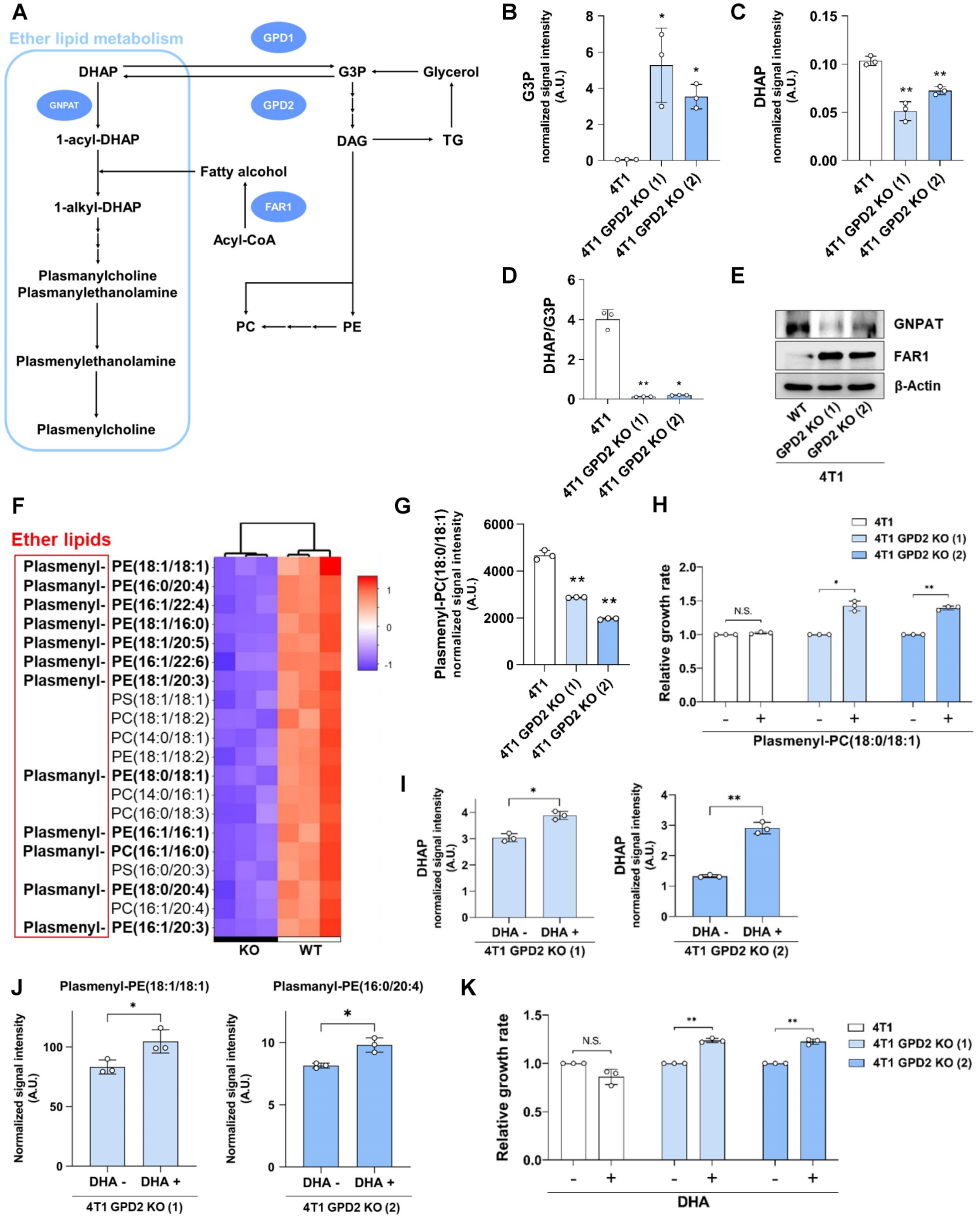
** 4T1 GPD2 KO cells exhibit downregulation of ether lipid synthetic pathway and plasmalogen level, and the cell growth is recovered by DHAP or plasmalogen supplementation. (A)** Scheme of ether lipid biosynthetic pathway and the related enzymes; GPD2, GNPAT, and FAR1. DAG: diacylglycerol. **(B)** Level of G3P in 4T1 and 4T1 GPD2 KO cells. **(C)** Level of DHAP in 4T1 and 4T1 GPD2 KO cells. **(D)** Cellular DHAP/G3P ratio for 4T1 and 4T1 GPD2 KO cells. For data (B-D), The signal intensities of DHAP and G3P were measured by LC-MS. **(E)** Expression of ether lipid synthetic pathway enzymes, GNPAT and FAR1, in 4T1 and 4T1 GPD2 KO cells, as detected by Western blot analysis. **(F)** Heatmap of top 20 most differentiated lipid species between 4T1 and 4T1 GPD2 KO (1) cell line. The “Plasmanyl-” prefix in the abbreviations represents ether lipids that have an ether bond at the *sn-1* position of the glycerol backbone, and the “Plasmenyl-” represents ether lipids that have a vinyl-ether bond at the *sn-1* position of the glycerol backbone, referred to as 'plasmalogens'. Ether lipids are indicated in bold. **(G)** Plasmalogen PC (18:0p/18:1) level in 4T1 and 4T1 GPD2 KO cells, as measured by LC-MS. **(H)** Relative growth rate of 4T1 and 4T1 GPD2 KO cells with or without plasmalogen PC (18:0p/18:1) treatment. The cell numbers of the plasmalogen-treated groups were normalized by paired untreated control groups. **(I)** Level of DHAP in 4T1 GPD2 KO cells with or without DHA treatment, as measured by LC-MS. **(J)** Levels of ether lipids in 4T1 GPD2 KO cells with or without DHA treatment, as measured by LC-MS. **(K)** Relative growth rate of 4T1 and 4T1 GPD2 KO cells with or without DHA treatment. The cell numbers of the DHA-treated groups were normalized by paired untreated control groups. Data were obtained from three biologically independent samples. The p-value was calculated by comparing the experimental group with 4T1 control group in data (B-D) and (G), and with paired untreated control groups in data (H-K), with two-tailed unpaired Student's t-test. The “*” in the graphs indicates statistically significant difference (“*”: p < 0.05; “**”: p < 0.005; “***”: p < 0.0005), and “N.S.,” 'not significant.' A.U., arbitrary unit.

For the next set of experiments, we conducted RNA-Seq using 4T1 and 4T1 GPD2 KO cells in an attempt to uncover the factors by which GPD2 affects tumor progression unrelatedly to bioenergetics. The RNA-Seq result showed little difference in carbohydrate metabolism (including glycolysis/gluconeogenesis, galactose, and pyruvate metabolism) or energy metabolism (including OXPHOS) **(Table 1),** consistent with the above-noted lack of any significant role of GPD2 in bioenergetics. On the other hand, we found a major difference in lipid metabolism, in particular in ether lipid metabolism and steroid biosynthesis **(Table 1).** Of these two pathways, ether lipid metabolism attracted our attention, since ether lipid synthesis starts from DHAP, which can be produced from G3P by a reaction catalyzed by GPD2 **(Figure 4A).** We therefore measured G3P and DHAP levels and compared the DHAP/G3P ratios. The 4T1 GPD2 KO cells had higher G3P level **(Figure 4B)** and lower DHAP level **(Figure 4C)**, resulting in much lower DHAP/G3P ratio **(Figure 4D),** as was the case also in 4T1 ρ0 cells **(*cf.* Figure 1E).** Consistently, overexpression of GPD2 in 4T1 cells shifted the DHAP-G3P equilibrium toward DHAP, increasing the DHAP/G3P ratio **(Figure S2).**

The relevance of GPD2 in the synthesis of ether lipids was further supported by evaluating key enzymes in the ether lipid biosynthetic pathway. The level of GNPAT, the first enzyme utilizing DHAP in the ether lipid biosynthetic pathway, was lower in GPD2 KO cells. In comparison, the level of FAR1, the rate-limiting enzyme in the ether lipid synthesis 60, was much higher in the KO cells **(Figure 4E).** It is noted that FAR1 protein level is primarily controlled via a negative feedback loop by cellular ether lipid level 61. Therefore, this result indicates a lack of feedback inhibition of FAR1 expression as a result of ether lipid depletion in GPD2 KO cells.

Difference in ether lipid levels between WT and GPD2 KO cells was investigated using tandem MS. In the untargeted LC-MS lipidomic analysis, ether lipids (plasmanyl-), including plasmalogens (plasmenyl-), were the most prevalent lipid species among the top 20 significantly biased lipid species in the KO cells **(Figure 4F),** while total TG and PC levels did not decrease **(Figure S3).** For more accurate quantification, we evaluated the level of a commercially available plasmalogen standard compound, plasmalogen PC (18:0p/18:1), by means of multiple reaction monitoring (MRM)-MS. As a result, the plasmalogen level was significantly lower in the KO cells compared to their WT counterparts **(Figure 4G).** These data suggest that GPD2 deficiency leads to a lower DHAP/G3P ratio that, in turn, promotes a decrease in plasmalogen level. To directly verify the hypothesis that lower level of DHAP, and its product plasmalogens, is responsible for the slower growth of cancer cells, we supplemented GPD2 KO cells with plasmalogen PC (18:0p/18:1), which was lower in the KO. The plasmalogen supplementation increased the growth of the KO cells by about 40%, with no effect on WT cells **(Figure 4H).** When supplementation of dihydroxyacetone (DHA), a cell-permeable precursor of DHAP 62, significantly increased the DHAP level **(Figure 4I),** and more importantly, the 12 different ether lipid species **(Figures 4J and S4)** which were downregulated in the KO cells **(*cf.* Figure 4F),** the DHA supplementation also increased the growth rate of KO cells, while it did not affect the growth of parental 4T1 cells **(Figure 4K).** These results confirm that GPD2 is a mediator for DHAP production and that DHAP and its product, plasmalogens, are involved in the regulation of cancer progression by GPD2.

### Ether lipid metabolism is linked to PI3K/Akt pathway

**Figure 5:**
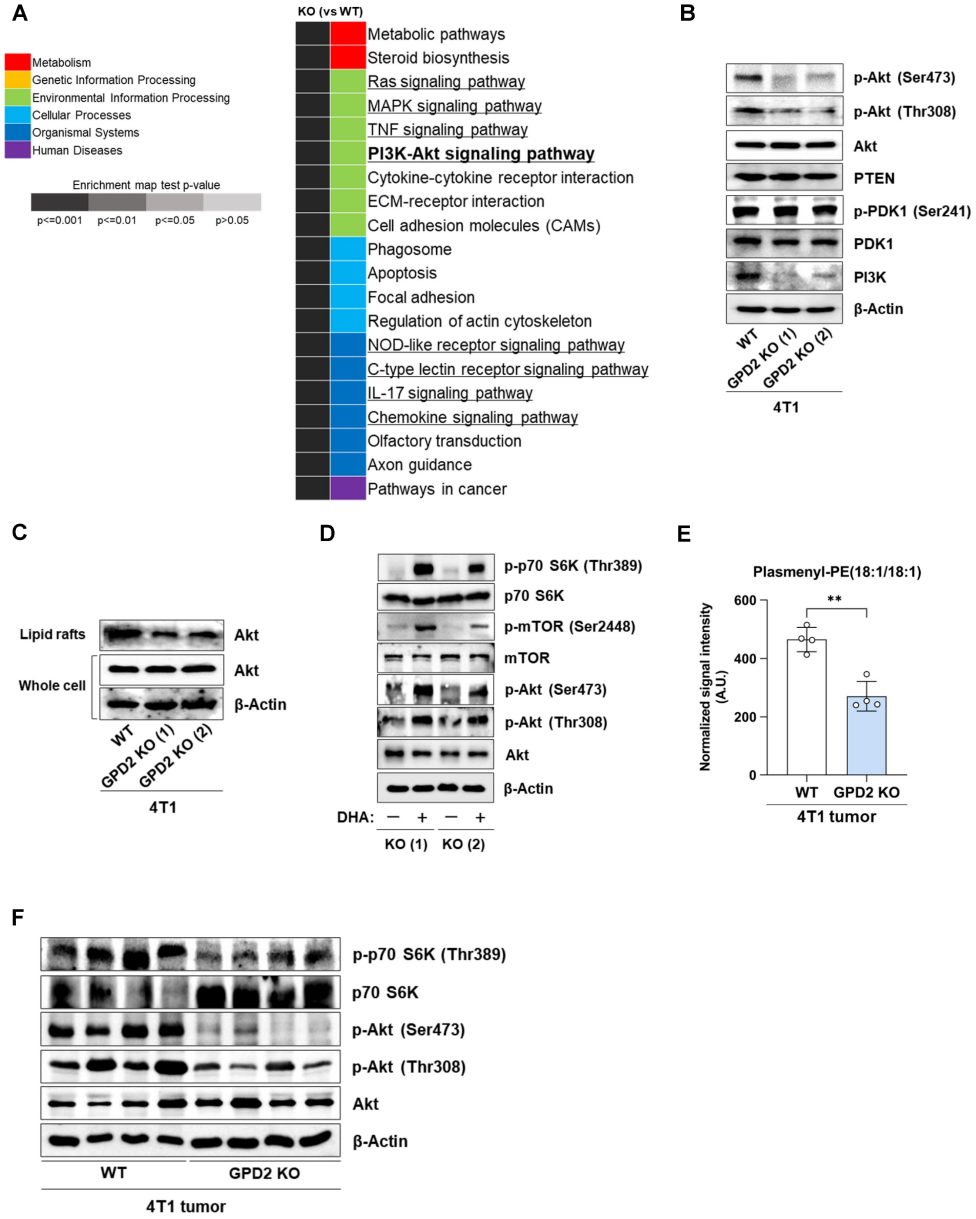
** GPD2 KO downregulates Akt signaling pathway, and it is recovered by DHAP supplementation. (A)** List of top 20 significantly enriched pathways in 4T1 GPD2 KO (1) cell line from GSEA with KEGG pathway. The signaling pathways are marked with underlines. **(B)** Expression of PI3K/Akt signaling pathway components in 4T1 and 4T1 GPD2 KO cells, as detected by Western blot analysis. **(C)** Expression of total Akt in the whole cell and the isolated lipid rafts of 4T1 and 4T1 GPD2 KO cells, as detected by Western blot analysis. **(D)** Expression of Akt/mTORC1 signaling pathway components with or without DHA treatment, as detected by Western blot analysis. **(E)** Level of ether lipids in WT and GPD2 KO of 4T1 graft tumor tissues, as measured by LC-MS. **(F)** Expression of Akt/mTORC1 signaling pathway components in WT and GPD2 KO of 4T1 graft tumor tissues. Data were obtained from three biologically independent samples or four graft tumors in each group unless indicated otherwise. The p-value was calculated by comparing the experimental group with 4T1 control group using a two-tailed unpaired Student's t-test. The “*” in the graphs indicates statistically significant difference (“*”: p < 0.05; “**”: p < 0.005; “***”: p < 0.0005). A.U., arbitrary unit.

To understand how plasmalogens may modulate cancer growth, we re-analyzed the RNA-Seq data using the GSEA with KEGG pathway approach. Of the top 20 most significantly affected pathways in GPD2 KO cells, eight of them were involving signaling pathways **(Figure 5A).** Of these, we focused on PI3K/Akt signaling pathway, since there are reports pointing to a relationship between plasmalogens and Akt activation in normal neuronal systems 63, 64. The GPD2 KO cells with lower plasmalogen ether lipids exhibited lower Akt phosphorylation, at both Thr308 and Ser473 and lower PI3K level, while there were no changes in PDK1 phosphorylation, and PTEN and total Akt level **(Figure 5B).** As lipid rafts are enriched in plasmalogen ether lipids 65 and they are known to be important for the activation of Akt 63, 66, we also compared the recruitment of Akt to lipid rafts in GPD2 WT and KO cells. When the Akt level was measured in the isolated lipid rafts, its level was lower in the KO cells, even though the Akt level in the whole cells was not different in the WT and KO cells **(Figure 5C).** This data shows that Akt recruitment to the lipid rafts is impaired in the plasmalogen-deficient GPD2 KO cells. Since DHAP supplementation increased the growth of GPD2 KO cells, we also tested if this can rescue the suppression of the PI3K/Akt pathway. Treatment of DHA on GPD2 KO cells activated Akt and its downstream signaling pathway components, including mTOR and p70 S6K, as evidenced by the increase in the phosphorylated form of these proteins **(Figure 5D).** In connection with the Akt signaling, we also measured the changes in cell cycle status in GPD2 KO cells, and the KO cells exhibited cell cycle arrest at S phase **(Figure S5).** To see the *in vivo* relevance of the proposed GPD2-ether lipid-Akt axis, we measured the ether lipid level in the grafted tumor tissues by using LC-MS. As a result, the level of ether lipids, the final end metabolites by GPD2 reaction, was lower in the GPD2 KO tumor tissues **(Figures 5E and S6),** establishing the GPD2-ether lipid link *in vivo.* Furthermore, the level of phosphorylated Akt (p-Akt) and its downstream target, phosphorylated p70 S6K (p-p70 S6K), as assessed by Western blot, was also lower in the GPD2 KO tumor tissues **(Figure 5F).** Therefore, these data confirm GDP2-ether lipid-Akt axis *in vivo*. In conclusion, our data suggest a new GPD2-ether lipid-Akt axis, whereby mitochondrial GPD2 regulates cancer cell growth by regulating plasmalogen level with ensuing activation of the Akt pathway.

### Involvement of GPD2 in different types of cancer

**Figure 6:**
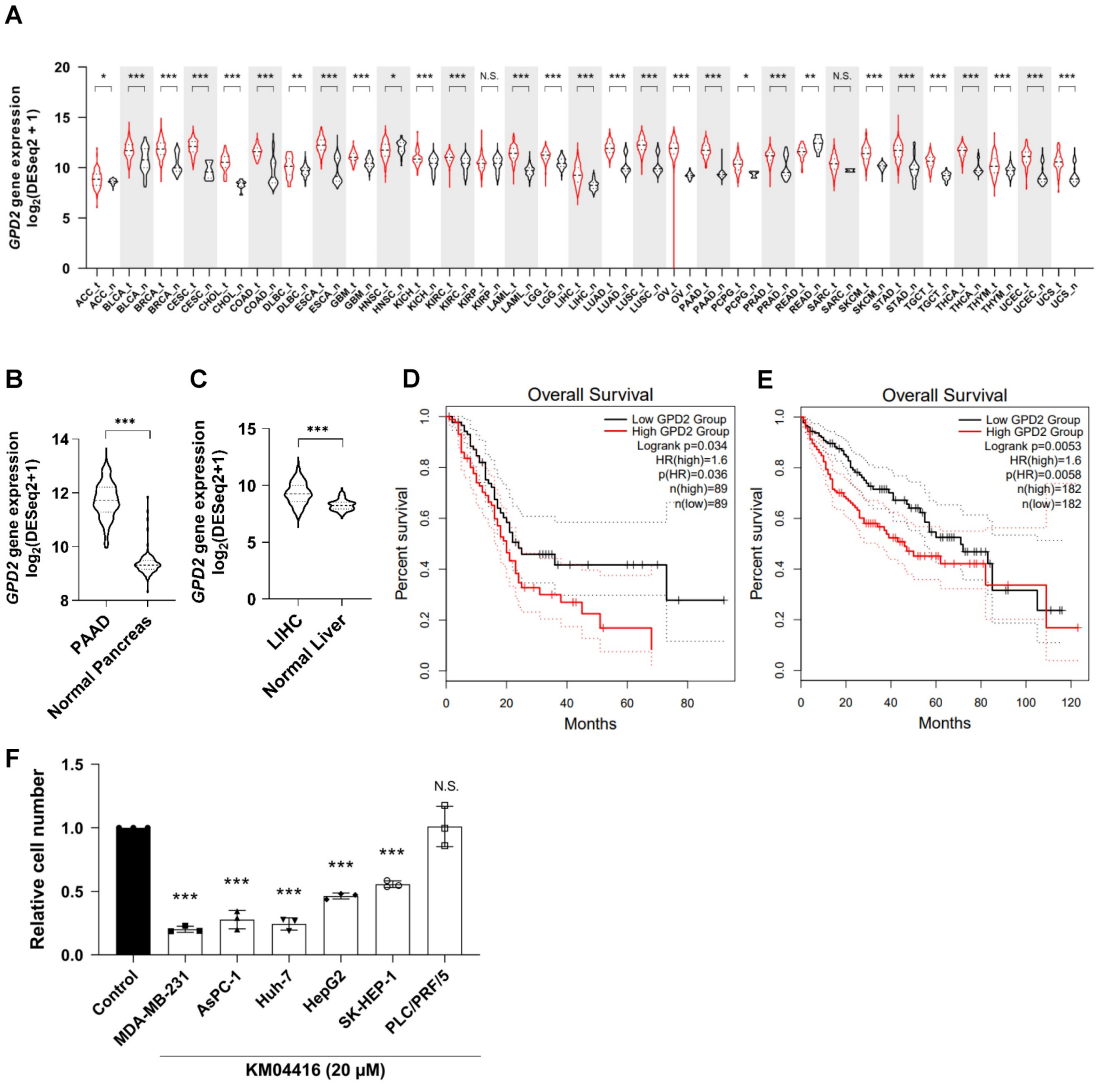
** GPD2 gene expression in various types of cancer and related patient survival. (A)** Gene expression distribution of *GPD2* among multiple tumor types and their normal counterparts. The gene expression data were downloaded from Xena (https://xenabrowser.net), and the classification of tumor-normal pairs was determined as described previously 54. The red lines represent tumor tissues, and the black lines, normal tissues. **(B)** Comparison of *GPD2* gene expression between samples from pancreatic adenocarcinoma (PAAD) and normal pancreas in (A). **(C)** Comparison of *GPD2* gene expression between samples from liver hepatocarcinoma (LIHC) and normal liver in (A).** (D)** Kaplan-Meier plot comparing overall survival of *GPD2*-high expression group (red line) and *GPD2*-low expression group (black line) in PAAD patients. **(E)** Kaplan-Meier plot comparing overall survival of *GPD2*-high expression group (red line) and *GPD2*-low expression group (black line) in LIHC patients. Survival analysis was performed in GEPIA 2 55 for data (D-E). **(F)** Relative cell number of various cancer cell lines with 20 µM KM04416 treatment for 48 h. The cell numbers of the KM04416-treated groups were normalized by paired untreated control groups. For data (A-C), the Wilcoxon rank-sum test was used to compare statistical significance between the groups. For data (D-E), the log-rank test was used to compare statistical significance between the groups. For data (F), the statistical significance was calculated by comparison with the control with two-tailed unpaired Student's t-test. The “*” in the graphs indicates statistically significant difference (“*”: p < 0.05; “**”: p < 0.005; “***”: p < 0.0005), and “N.S.,” 'not significant.'

Having uncovered a plausible mechanism by which GPD2 regulates cancer, we next explored the relevance of the enzyme in different types of human cancer. Transcriptomic comparison between cancer and normal tissues using the cancer gene atlas (TCGA) TARGET GTEx database showed that most of the human cancers evaluated (27 out of 31) exhibit significantly higher GPD2 expression than their normal counterparts **(Figure 6A),** leading to its higher median expression in cancer tissues over the entire cancer landscape in the datasets (n = 9793 for tumor and 4888 for normal tissue; **Figure S7A**). In addition, the overall survival rate for all cancer patients with higher GPD2 expression (n = 4747) was lower than for those with lower GPD2 expression (n = 4747) **(Figure S7B).** For an example, expression of GPD2 gene was higher in pancreatic **(Figure 6B)** and liver cancer tissues **(Figures 6C)** than in normal counterparts, with correspondingly worse survival prognosis for patients in the GPD2-high groups **(Figures 6D and E, respectively).** The differential expression between normal and tumor tissues for liver and pancreas was independently confirmed at the protein level using the CPTAC proteomics database (**Figures S7C and D**). On behalf of the database, we also observed a growth inhibitory effect of the GPD2 inhibitor, KM04416, on several cancer cell lines including MDA-MB-231 (breast cancer) and AsPC-1 (pancreatic cancer) as well as Huh-7, HepG2, and SK-HEP-1 cells (liver cancer), except for PLC/PRF/5 cells (human hepatoma cell line) **(Figure 6F)**. Therefore, the role of GPD2 may be implicated over a wider range of human cancers, making the enzyme a promising target for cancer therapy.

## Discussion

Mitochondria are essential for proper function of not only normal cells but also cancer cells. In our previous study with the mtDNA-deficient ρ0 cancer cell model with extensive mitochondrial damage 52, we found that functional mitochondrial respiration is important in tumorigenesis for maintaining DHODH-driven pyrimidine biosynthesis rather than for ATP production. This link between pyrimidine synthesis and respiration showed an intriguing interplay between mitochondrial bioenergetics and biosynthesis. Since the complex roles of mitochondria in cancer cannot be explained by involvement of a single protein, we employed the same ρ0 cancer cell model using different approaches to find additional player(s) in mitochondria that contribute to cancer growth. Our study revealed that the DHAP/G3P ratio regulated by GPD2 affects plasmalogen lipid synthesis that, in turn, regulates important signaling pathways in cancer. Interestingly, a related cytosolic enzyme, GPD1, expression was not different between the parental and ρ0 cells. Consistently, recent studies showed that only GPD2 inhibition, but not GPD1 overexpression, increased G3P concentration in several cancer cells 67, 68. These results reveal that mitochondria-localized GPD2 has a specific function of mediating the interplay between lipid biosynthesis and cancer cell signaling. Therefore, the examples of DHODH and GPD2 demonstrate the importance of mitochondria-driven biosynthesis in cancer biology along with mitochondria's established bioenergetic, epigenetic, and catabolic roles. This biosynthetic role could also be a druggable vulnerability for cancer. While DHODH is an essential and ubiquitous enzyme for both normal and cancer cell growth, and its KO is embryonically lethal, GPD2 KO is not devastating, and exhibits normal liver and muscle metabolism in adult mice, albeit causing reduced viability (~50%) of pups 69. As GPD2 elevation was also demonstrated in many fast-growing tumors 70, 71, GPD2 may be a new mitochondrial target for cancer cell metabolism.

GPD2 is at the crossroad of several important cell metabolism processes: gluconeogenesis in glucose metabolism, phospholipid and TG biosynthesis in lipid metabolism, and electron transfer in mitochondrial OXPHOS 72. The roles of GPD2 in cancer cell growth, however, have not been well defined. A study on prostate cancer cells proposed GPD2 as an ROS producer in cancer progression 57. Other studies have implicated GPD2 in different bioenergetic roles such as OXPHOS regulation in thyroid cancer 25, glycolysis regulation in glioma and liver cancer 26, 73, and G3P-driven ATP synthesis activation 27. However, there are also contradictory studies to the ROS or bioenergetics-related roles. As for the ROS-related roles, one study showed higher G3P-dependent ROS production in GPD2-low tissues 18. In addition, the presence of many other ROS-producing dehydrogenases, such as α-ketoglutarate dehydrogenase 74 and ETF dehydrogenase 75, diminishes the role for GPD2, if any, as a major ROS-producing enzyme. This is also supported by the lack of difference in mitochondrial superoxide or total ROS level between WT and GPD2 KO cells of our model. Concerning studies on bioenergetic roles, no change in the energy status was observed following GPD2 knockdown, in another study 20. Moreover, the levels of various energy-linked metabolites including ATP, AMP, pyruvate, and lactate did not differ between GPD2 WT- and KO-adult mice 69. We also obtained consistent results showing no changes in ATP level, lactate production and energy status in GPD2 KO cells. Furthermore, the relative portion of GPD2-dependent respiration was much lower than those of the other respiratory complexes. Taken together, the previous relevant results and our present results argue against GPD2's major roles in regulating mitochondrial bioenergetics or ROS in the context of cancer cell growth.

On the other hand, increased cell growth by supplementation of either DHAP precursor or plasmalogen in GPD2 KO cells points to ether lipid biosynthesis as the primary role of GPD2 in cancer cell growth. Whereas GPD2's main function is to convert G3P to DHAP, there may be other sources of DHAP, such as glycolysis, since GPD2 KO was not able to completely deplete plasmalogens. It should be also noted that the KO cells had a lower steady-state plasmalogen level and grew slower than the parental cells, despite a sufficient glucose supply. This suggests that GPD2-derived DHAP has a significant role in plasmalogen synthesis even when other possible sources are functioning properly. As ether lipid synthesis is known to take place mostly in peroxisomes 76, 77, both glycolysis-derived and mitochondria-derived (via GPD2) DHAP need to be transported to these organelles. For cytosolic DHAP, there may a yet-to-be discovered transport mechanism, as DHAP by itself is membrane-impermeable. For mitochondrial DHAP, it may be transferred via the well-known direct contact sites between the two organelles 14, 15. Direct organelle contacts are also important for transport of ether lipids from peroxisomes to mitochondria via the endoplasmic reticulum 78. Quantification of the respective contributions of mitochondria versus cytosolic DHAP to ether lipid synthesis should be an interesting topic for future studies. It is also worth noting that GPD2 KO increased the total PC level, which can be explained by the shifting of the DHAP-G3P equilibrium toward G3P. Overall, GPD2 is suggested to be a key regulator at the ether lipid-phospholipid branchpoint by shifting the equilibrium between DHAP and G3P through DHAP/G3P ratio.

Ether lipids have been implicated in various pathophysiological states, such as neural and metabolic diseases 33, 79. Most often, the ether lipid level was lower in disease cases than in normal ones. However, the ether lipid level in cancers was found to be elevated relative to normal tissues 43, 45, 46, which is consistent with the higher level and activity of GPD2 in many types of cancer. Nevertheless, the involvement of ether lipids in cancer has been mostly anecdotal, and its mechanistic contribution to tumor growth has remained unknown. In this respect, an important finding by us is that GPD2-mediated ether lipid biosynthesis activates the Akt signaling pathway, which suggests that ether lipids present a mechanistic link between GPD2 and the Akt pathway in cancer cells. This proposition is consistent with the above-noted elevated levels of ether lipids in various cancers, particularly as Akt signaling is frequently activated in the context of tumor progression. Interestingly, the link between ether lipids and the Akt pathway has been shown for normal neural cells. In Schwann cells, ether lipids have been found to be critical to differentiation and myelination via Akt activation and its downstream suppression of GSK3β 63. Also, plasmalogens have been reported to promote PI3K-dependent Akt phosphorylation and to protect neuronal cells from cell death induced by stress conditions 64.

Therefore, our present results can be considered to extend the ether lipid-Akt link to non-neuronal, cancer cells and to associate it with GPD2. A natural question in this regard is how ether lipids activate the Akt pathway. It has been reported that lipid rafts in plasma membrane are enriched in plasmalogens 65, and the lipid rafts are essential for the spatial compartmentalization of components for activating PI3K/Akt signaling pathway 66. This has also been addressed in a study for neurons, wherein ether lipids form lipid raft-type microdomains and promote activation of G-protein coupled receptors, thus facilitating membrane recruitment of Akt and triggering its subsequent phosphorylation 80. Our results show that, while there were no changes in PDK1 and PTEN, the plasmalogen deficiency still decreased the Akt phosphorylation, at both Thr308 and Ser473. The same phenomenon has been reported in schwann cells, suggesting the necessity of plasmalogens for the right compartmentalization and phosphorylation of Akt 63. This may be due to the decreased lipid raft surface area or altered lipid raft composition because of the plasmalogen deficiency. More studies are warranted for detailed mechanism of lipid raft-associated Akt activation. We studied the PI3K/Akt pathway as a downstream of the GPD2-DHAP-ether lipid axis based on currently available literature. The ether lipid-Akt relationship has been known only in normal neuronal cells, not in cancer cells. We also found that the ether lipid-Akt axis is regulated by GPD2-generated DHAP. As the membrane composition of cancer cells and neurons differs, we do not exclude the possibility of another mechanism through which plasmalogens promote the Akt pathway and cancer cell growth, including other pathways involved in Figure 5A. For example, ether lipids have been shown to scavenge ROS 81-83, which might help cancer cells to evade ROS-induced damage. This should be an interesting topic for future research.

We also observed activation of Akt downstream components, such as mTORC1 and S6K, as a consequence of DHAP precursor supplementation in GPD2 KO cells. Although mTORC1 is known to be regulated either in an AMPK-dependent 84 or independent manner 85-87 in terms of glucose sensing, precisely how AMPK-independent regulation is orchestrated is still not well understood 88, 89. Recently, Orozco and colleagues suggested glucose-derived DHAP as a key molecule for AMPK-independent mTORC1 activation 62, but the exact molecular mechanism linking DHAP and mTORC1 remained unexplained. We have demonstrated here downregulation of the Akt pathway by GPD2 KO, the recovery of Akt/mTORC1 activation by DHAP precursor and ether lipid supplementation, and lower levels of ether lipids in DHAP-low GPD2 KO cells. These findings may be interpreted as pointing to DHAP-driven ether lipid biosynthesis as the missing link between DHAP and mTORC1 activation. Notwithstanding the notion that further studies are needed to unravel the details of this hypothesis, our study provides a novel role for GPD2 in cancer unrelated to its involvement in bioenergetics, pointing to its key role in anabolic processes.

**Figure 7:**
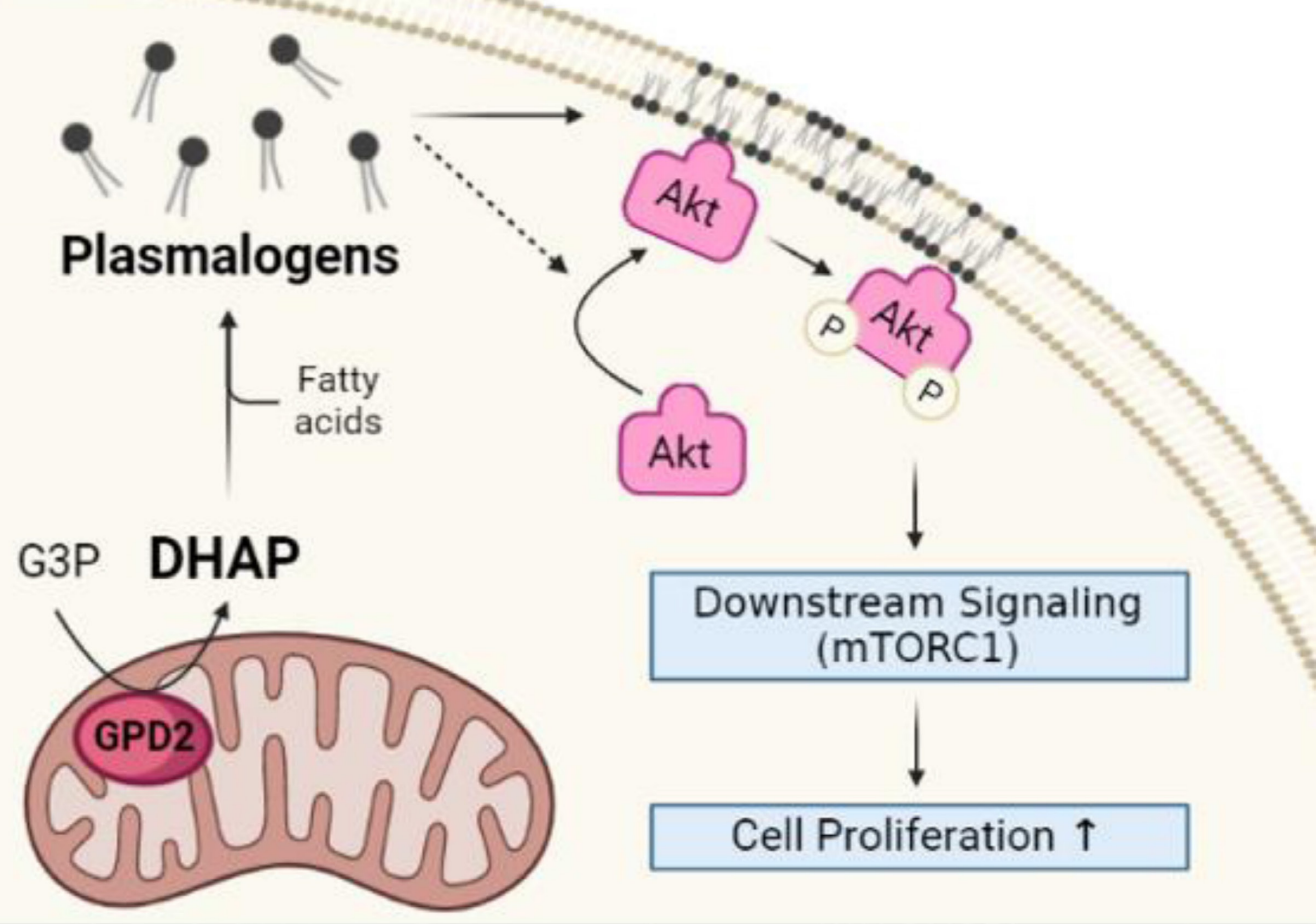
**Schematic of GPD2-ether lipid-Akt axis in cancer growth control.** GPD2 produces DHAP from G3P. DHAP is then used as a building block for plasmalogen synthesis. The synthesized plasmalogens form lipid rafts in the plasma membrane, and compartmentalize Akt within the rafts. After Akt is recruited to the membrane, it is phosphorylated and activates the downstream signaling, including mTORC1. The activation of Akt signaling pathway induces cancer cell growth. The illustration was created with BioRender.com.

In conclusion, our findings on non-bioenergetic role of mitochondrial GPD2 in the regulation of DHAP production and ether lipid synthesis, and the activation of PI3K/Akt signaling pathway as the downstream of the GPD2-DHAP-ether lipid axis suggest 'GPD2-ether lipid-Akt axis' as an anabolic role of mitochondria in cancer growth **(Figure 7).** Based on these, it is believed that GPD2-mediated ether lipid biosynthesis is a vulnerability in cancer. Although current GPD2 inhibitors, such as KM04416 used in our experiment, are not potent enough to be used in clinics, studies such as ours are expected to motivate research toward more potent GPD2 inhibition therapy that may find clinical use. Future development of more efficient and specific inhibitors may lead to better options for targeting of GPD2.

## Supplementary Material

Supplementary figures.

## Figures and Tables

**Table 1:** Changes in overall metabolism by GPD2 KO in 4T1 cancer cells as analyzed by RNA-Seq

Metabolism	Pathway	p-value
Carbohydrate metabolism	*Glycolysis / Gluconeogenesis*	0.34
	*Pentose phosphate pathway*	0.44
	*Pentose and glucuronate interconversions*	1
	*Fructose and mannose metabolism*	*
	*Galactose metabolism*	1
	*Ascorbate and aldarate metabolism*	1
	*Starch and sucrose metabolism*	1
	*Amino sugar and nucleotide sugar metabolism*	1
	*Pyruvate metabolism*	0.5
	*Glyoxylate and dicarboxylate metabolism*	1
	*Propanoate metabolism*	0.45
	*Butanoate metabolism*	0.39
	*Inositol phosphate metabolism*	*
Carbohydrate metabolism	*Oxidative phosphorylation*	0.52
	*Nitrogen metabolism*	0.27
	*Sulfur metabolism*	1
Lipid metabolism	*Fatty acid biosynthesis*	1
	*Fatty acid degradation*	0.6
	*Synthesis and degradation of ketone bodies*	0.19
	*Steroid biosynthesis*	***
	*Primary bile acid biosynthesis*	*
	*Steroid hormone biosynthesis*	0.08
	*Glycerolipid metabolism*	0.1
	*Glycerophospholipid metabolism*	**
	*Ether lipid metabolism*	***
	*Sphingolipid metabolism*	0.06
	*Arachidonic acid metabolism*	*
	*Linoleic acid metabolism*	*
	*alpha-Linolenic acid metabolism*	*

“*”: p < 0.05; “**”: p < 0.005; “***”: p < 0.0005.

## Abbreviations

AGPS: alkylglycerone phosphate synthase
CCCP: carbonyl cyanide m-chlorophenyl hydrazone
CI: mitochondrial respiratory complex I
CII: mitochondrial respiratory complex II
CoQ: coenzyme Q
Cyt C: cytochrome c
DEG: differentially expressed genes
DHA: dihydroxyacetone
DHAP: dihydroxyacetone phosphate
ETC: electron transport chain
FAD: flavin adenine dinucleotide
FAR1: fatty acyl-CoA reductase 1
FH: fumarate hydratase
FPKM: fragments per kilobase of transcript per million mapped reads
G3P: glycerol-3-phosphate
GNPAT: glyceronephosphate O-acyltransferase
GPAT: glycerol-3-phosphate acyltransferase
GPD1: cytosolic glycerol-3-phosphate dehydrogenase
GPD2: mitochondrial glycerol-3-phosphate dehydrogenase
GPS: glycerol phosphate shuttle
GSEA: gene-set enrichment analysis
HSQC: heteronuclear single quantum coherence
IDH: isocitrate dehydrogenase
IMS: intermembrane space
KEGG: Kyoto encyclopedia of genes and genomes
KO: knockout
LC-MS: liquid chromatography-mass spectrometry
MRM: multiple reaction monitoring
mtDNA: mitochondrial DNA
NMR: nuclear magnetic resonance
OXPHOS: oxidative phosphorylation
p-Akt: phosphorylated form of Akt
PC: phosphatidylcholine
p-p70 S6K: phosphorylated form of p70 S6K
RNA-Seq: RNA sequencing
ROS: reactive oxygen species
SDH: succinate dehydrogenase
TCA: tricarboxylic acid
TCGA: the cancer gene atlas
TG: triglyceride
VDAC: voltage-dependent anion channel
WT: wildtype
ρ0: mitochondrial DNA-deficient
