# Supplementary material for: Non-bioenergetic roles of mitochondrial GPD2 promote tumor progression
Source: Theranostics. 2023 Jan 1;13(2):438–57. doi: 10.7150/thno.75973 (PMC9830446; doi:10.7150/thno.75973)
Supplement: Supplementary file 1 — Supplementary figures. [file thnov13p0438s1.pdf]

Non-bioenergetic roles of mitochondrial GPD2 promote tumor progression

Sehyun Oh[1], Sihyang Jo[1], Martina Bajzikova[2], Han Sun Kim[1], Thien T. P. Dao[1], Jakub Rohlena[4], Jin-Mo Kim[1], Jiri Neuzil[*,2,4,5], Sunghyouk Park[*,1]

[1]College of Pharmacy, Natural Product Research Institute, Seoul National University, Seoul 08826, Korea
[2]School of Pharmacy and Medical Science, Griffith University, Southport, Qld, Australia
[3]Institute of Physiology, Czech Academy of Sciences, Prague, Czech Republic
[4]Institute of Biotechnology, Czech Academy of Sciences, Prague-West, Czech Republic
[5]Faculty of Science, Charles University, Prague, Czech Republic

*Corresponding Authors
Sunghyouk Park
Natural Product Research Institute, College of Pharmacy, Seoul National University, Gwanak-Ro 1, Gwanak-gu, Seoul 08826, Republic of Korea, Tel: +82-2-880-7831; Fax: +82-2-880-7831; E-mail: psh@snu.ac.kr
Jiri Neuzil
School of Pharmacy and Medical Science, Griffith University, 1 Parklands Dr, Southport, Qld 4215, Australia, Tel: +61-(0)7-5552-9109; Fax: +61-(0)7-5552-9109; E-mail: j.neuzil@griffith.edu.au or jiri.neuzil@ibt.cas.cz

Supplementary Information

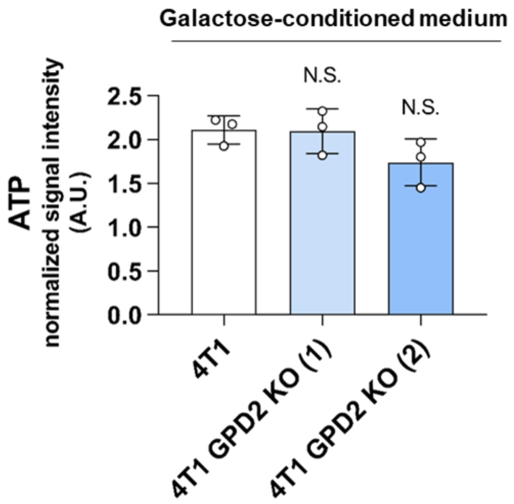

Supplementary Figure 1: ATP level of 4T1 and 4T1 GPD2 KO cells grown in galactose-conditioned medium. The signal intensity was obtained by LC-MS and normalized by BCA value. Data were obtained from three biologically independent samples. The p-value was calculated by comparing the experimental group with 4T1 control group with two-tailed unpaired Student’s t-test. The “*” in the graphs indicates statistically significant difference (“*”: $p < 0.05$; “**”: $p < 0.005$; “***”: $p < 0.0005$), and “N.S.,” ‘not significant.’ A.U., arbitrary unit

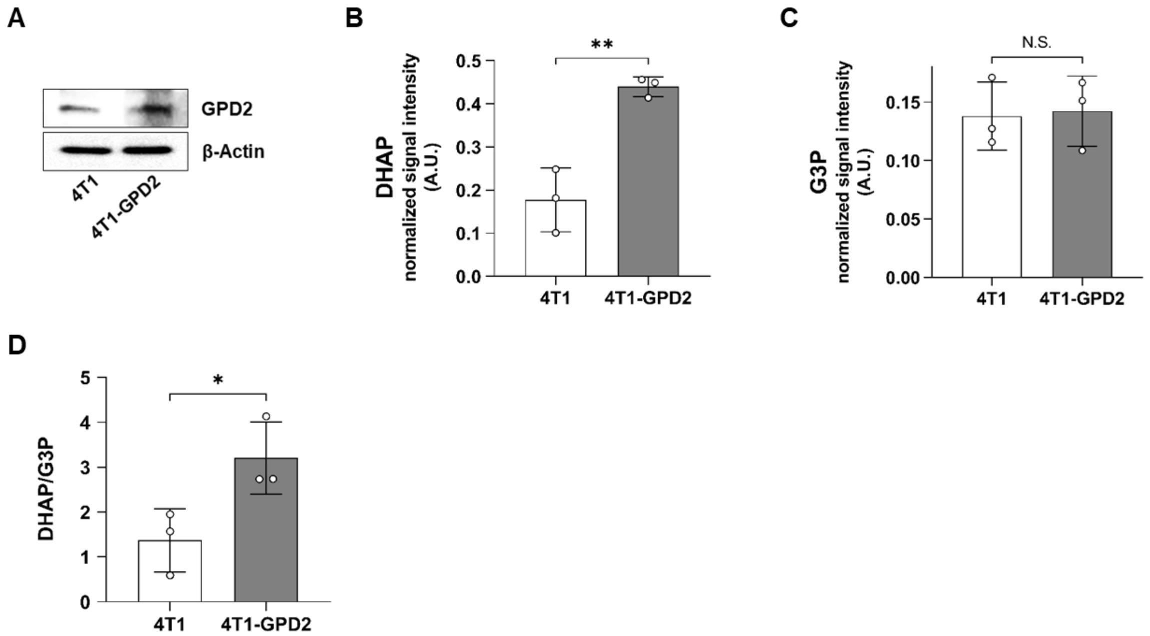

Supplementary Figure 2: DHAP/G3P ratio in 4T1 cells with GPD2 overexpression. (A) Protein expression of GPD2 in 4T1 and 4T1-GPD2 (GPD2 overexpression) cells as detected by Western blot analysis. **(B)** Level of DHAP in 4T1 and 4T1-GPD2 cells. **(C)** Level of G3P in 4T1 and 4T1-GPD2 cells. **(D)** Cellular DHAP/G3P ratio in 4T1 and 4T1-GPD2 cells.

In data (B-D), the signal intensities were obtained by LC-MS and normalized by BCA value. Data were obtained from three biologically independent samples. The p-value was calculated by comparing the experimental group with 4T1 control group with two-tailed unpaired Student's t-test. The "*" in the graphs indicates statistically significant difference ("*": $p < 0.05$; "**": $p < 0.005$; "***": $p < 0.0005$), and "N.S.," 'not significant.' A.U., arbitrary unit

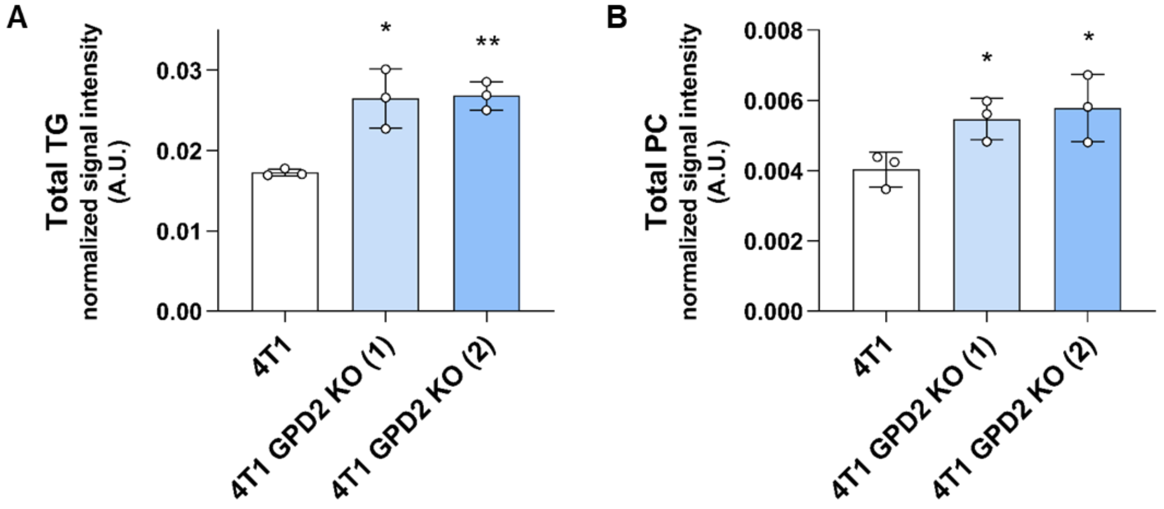

Supplementary Figure 3: Level of total TG and PC in 4T1 and 4T1 GPD2 KO cells. (A) TG level in 4T1 and 4T1 GPD2 KO cells. **(B)** PC level in 4T1 and 4T1 GPD2 KO cells. The signal intensities were obtained by NMR and normalized by BCA value.

Data were obtained from three biologically independent samples. The p-value was calculated by comparing the experimental group with 4T1 control group with two-tailed unpaired Student’s t-test. The “*” in the graphs indicates statistically significant difference (“*”: $p < 0.05$; “**”: $p < 0.005$; “***”: $p < 0.0005$). A.U., arbitrary unit

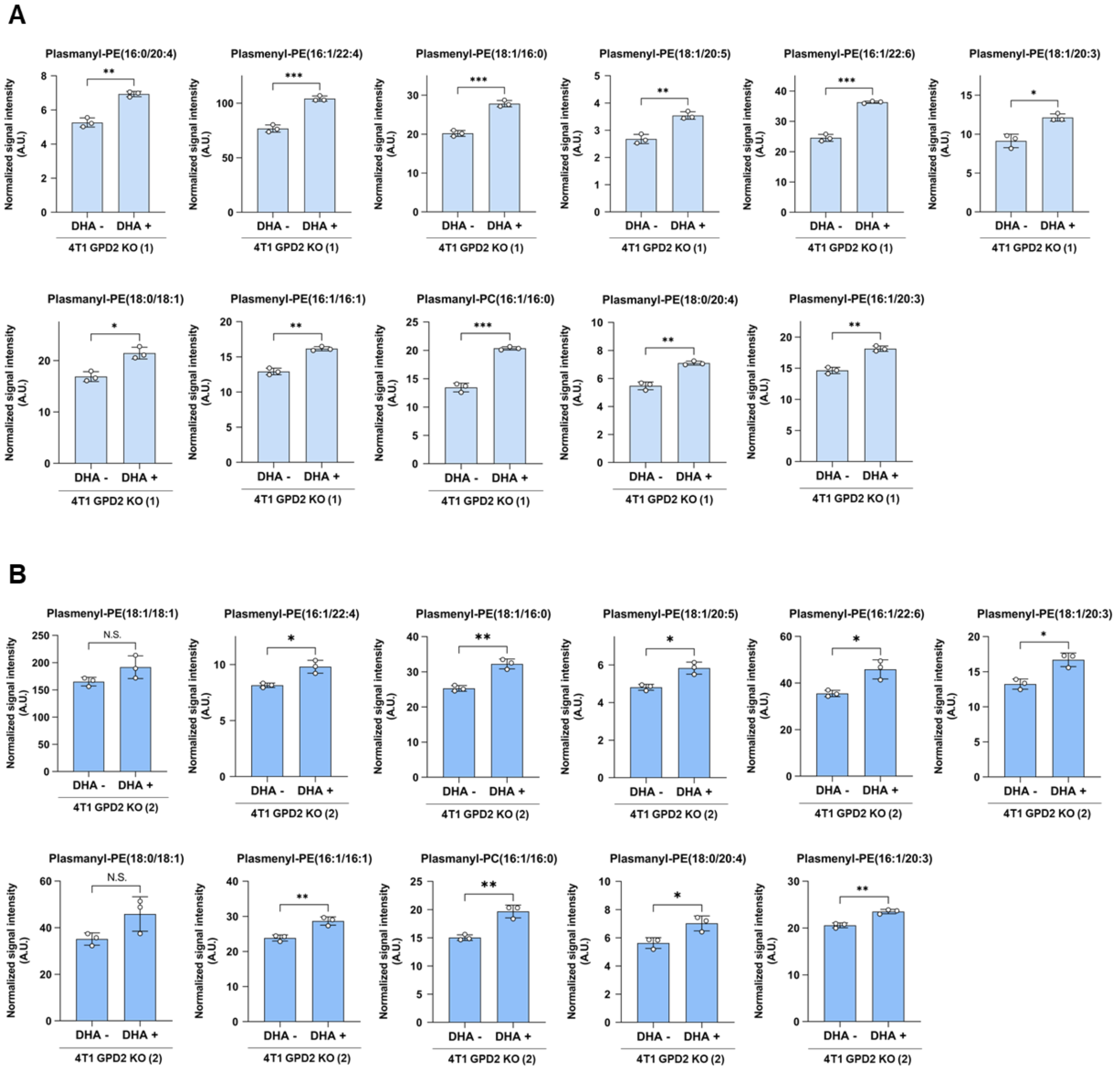

Supplementary Figure 4: Levels of different ether lipid species in 4T1 GPD2 KO cells with or without DHA treatment. (A) Ether lipid level with or without DHA treatment in 4T1 GPD2 KO (1) cells. **(B)** Ether lipid level with or without DHA treatment in 4T1 GPD2 KO (2) cells.

In data (A-B), the signal intensity was obtained by LC-MS and normalized by BCA value. Data were obtained from three biologically independent samples. The p-value was calculated by comparing the experimental group with 4T1 control group with two-tailed unpaired Student's t-test. The "*" in the graphs indicates statistically significant difference ("*": $p < 0.05$; "**": $p < 0.005$; "***": $p < 0.0005$), and "N.S.," 'not significant.' A.U., arbitrary unit

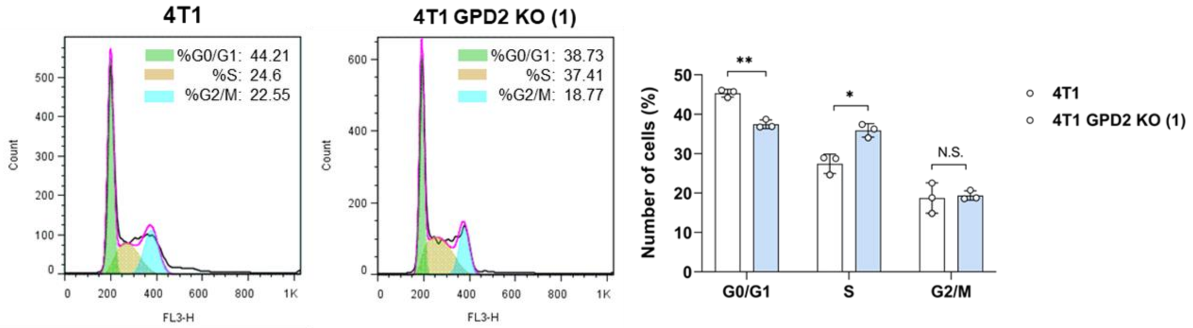

Supplementary Figure 5: Representative histogram and bar graph of cell cycle progression in 4T1 and 4T1 GPD2 KO cells. Data were obtained from three biologically independent samples. The p-value was calculated by comparing the experimental group with 4T1 control group with two-tailed unpaired Student's t-test. The "*" in the graphs indicates statistically significant difference ("*": $p < 0.05$; "**": $p < 0.005$; "***": $p < 0.0005$), and "N.S.," 'not significant.'

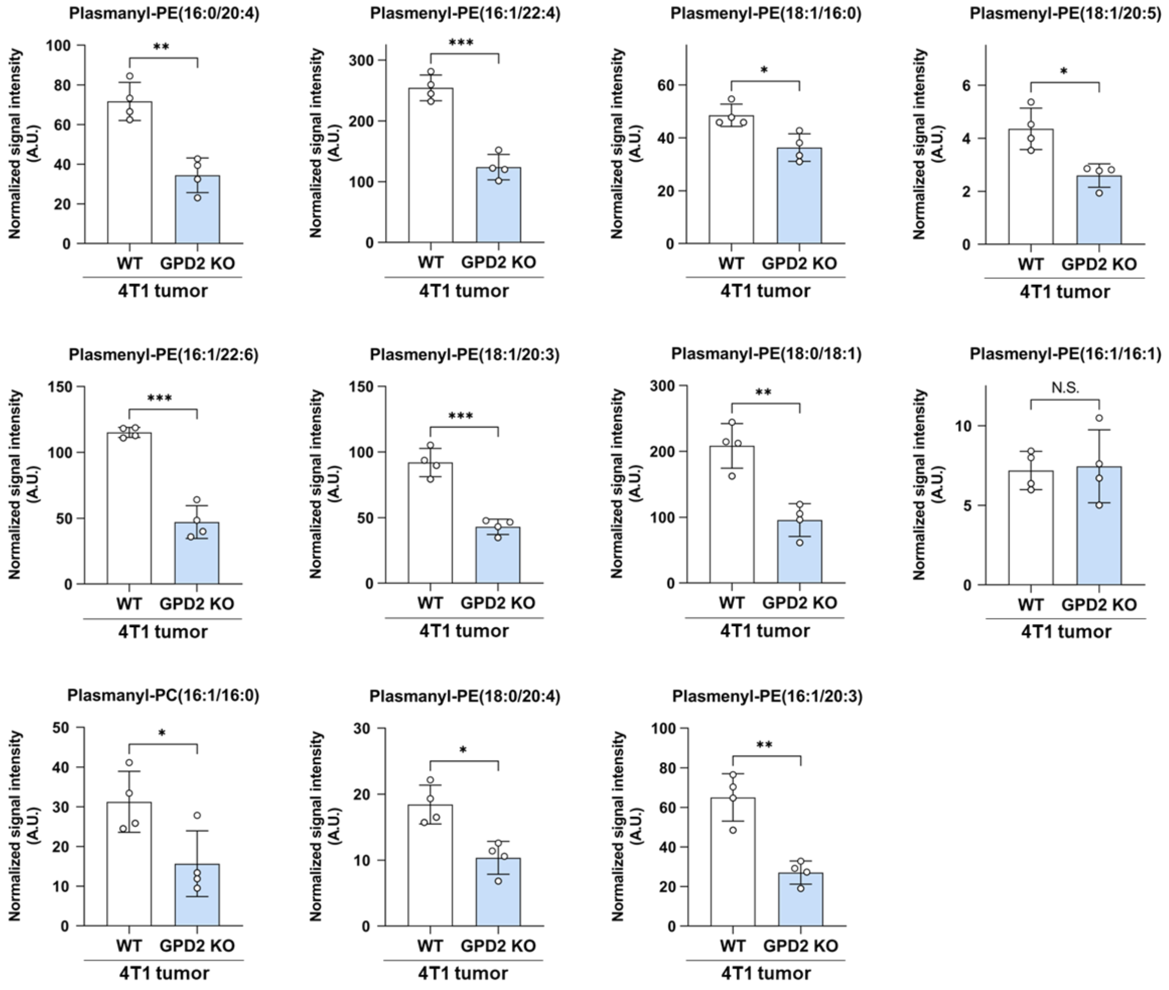

Supplementary Figure 6: Levels of different ether lipid species in WT and GPD2 KO of 4T1 graft tumor tissues. The signal intensities were obtained by LC-MS and normalized by BCA value. Data were obtained from four biologically independent samples. The p-value was calculated by comparing the experimental group with 4T1 control group with two-tailed unpaired Student's t-test. The "*" in the graphs indicates statistically significant difference ("*": $p < 0.05$; "**": $p < 0.005$; "***": $p < 0.0005$), and "N.S.," 'not significant.' A.U., arbitrary unit

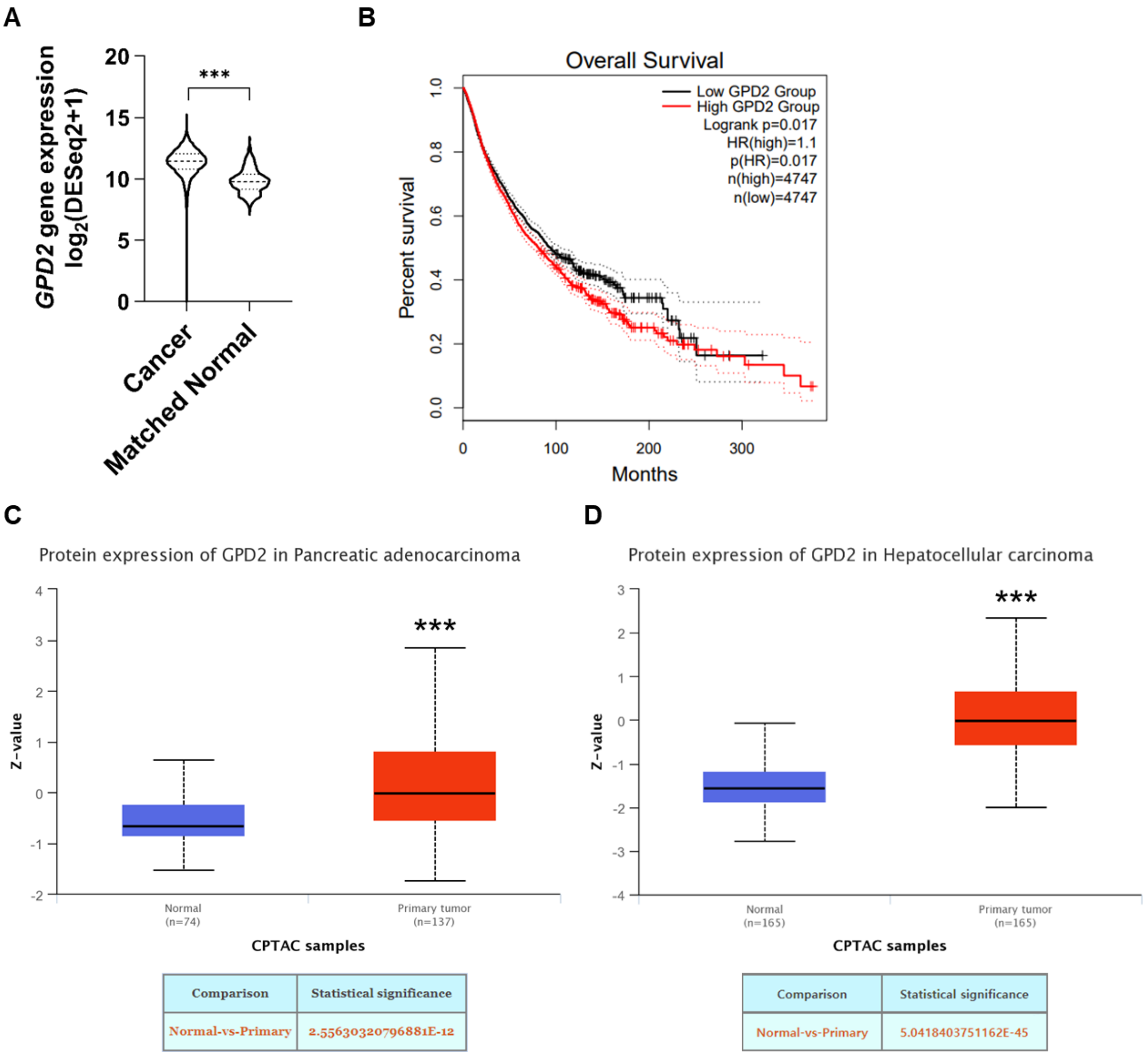

Supplementary Figure 7: GPD2 expression in various types of cancer and related patient survival. (A) Comparison of *GPD2* gene expression between samples from all cancer tissue types and their normal counterparts in Figure 6A. For those in normal tissues, duplicate samples were excluded. **(B)** Kaplan-Meier plot comparing overall survival of *GPD2*-high expression group (red line) and *GPD2*-low expression group (black line) in patients for all cancer tissue types. Survival analysis was performed in GEPIA 2 (http://gepia2.cancer-pku.cn) [55]. **(C-D)** GPD2 protein level comparison between normal and cancer tissues for pancreatic adenocarcinoma (C) and liver cancer (D) from CPTAC proteomic database**.**

For data (A), the Wilcoxon rank-sum test was used to compare statistical significance between the groups. For data (B), the log-rank test was used to compare statistical significance between the groups. For data (C-D), the Student's t-test was used to compare statistical significance between the groups. The "*" in the graphs indicates statistically significant difference ("*": $p < 0.05$; "**": $p < 0.005$; "***": $p < 0.0005$).
